# Demographic and health impacts of women’s bodily autonomy: switching prescription requirements for emergency contraceptives

**DOI:** 10.1007/s10198-025-01863-7

**Published:** 2025-11-20

**Authors:** Fabian Grünwald, Tom Stargardt

**Affiliations:** Hamburg Center for Health Economics, University of Hamburg, Hamburg, Germany

**Keywords:** Emergency contraception, OTC switch, Abortions, Birth rate, Sexually transmitted diseases, I12, I18, J13, J18

## Abstract

Over the past decades, most European countries switched emergency contraceptive availability from prescription to over-the-counter (OTC) status. However, the effect of OTC switches on emergency and regular contraceptive use, on demographic indicators (birth rate, abortion rate) and health outcomes (sexually transmitted diseases) varies depending on the study setting. Only Poland switched emergency contraceptives back to prescription (Rx) status after previously loosening restrictions and causal analyses of an Rx switch are lacking entirely. We employ multiple (staggered/synthetic) difference-in-differences models to evaluate the effect of (a) an OTC switch, exploiting variation in the timing of the OTC switch among several European countries and (b) a Rx switch at the example of Poland. We find that the use of emergency contraceptives increased (decreased) after OTC (Rx) switches. The use of regular hormonal contraceptives, the rate of births and abortions and the incidence of various sexually transmitted diseases remained essentially unaffected or – if found - effects did not withstand robustness checks. Our results suggest that increased use of emergency contraceptives due to liberalized over-the-counter access may be reversed by a return to prescription bound access.

**Supplementary Information:**

The online version contains supplementary material available at 10.1007/s10198-025-01863-7.

## Introduction

Recent protests in the USA (2022) and Poland (2021) against new, stricter abortion laws [1–3] and the U.S. Supreme Court decision to preserve access to the abortion pill mifepristone [4] have shown that women’s bodily and reproductive autonomy is still controversial. A closely related topic is the availability of emergency contraceptives. While abortions stop ongoing pregnancies, emergency contraceptives actually prevent pregnancies by delaying ovulation and must be taken shortly after intercourse [5].

Similar to abortions, access to these contraceptives has varied in Europe. Over time, most countries have voluntarily switched these medications from prescription (Rx) to over-the-counter (OTC) status (“OTC switch”), the latter of which means that they can be obtained from pharmacies without the need to consult a physician [6]. Although some member countries of the European Union (EU) did not follow this example, they were obliged to do so when the European Medicines Agency (EMA) itself switched the centrally approved emergency contraceptive ulipristal acetate to OTC status [7]. Hungary was an exception and refused to incorporate the EMA decision into national law. Poland accepted the EMA ruling at first but revoked the OTC status two years later and reinstated the prescription requirement [6, 8]. This switch back allows us to consider not only the OTC switch but also the opposite scenario in an additional analysis. We will refer to this as an “Rx switch”.

Most of the previous literature has linked simplified access to emergency contraceptives with increased use. The impact of simplified access on demographic indicators (e.g., birth rate, fertility, abortion rate), the incidence of sexually transmitted diseases (STIs) and changes in risk-taking behavior is more ambiguous, however, and the findings in this regard depend on the study setting, such as which countries and subpopulations are observed, as most existing studies focus on single countries, and the circumstances of emergency contraceptive provision [6, 7, 9–21]. To the best of our knowledge, no causal study has analyzed the switch in the status of emergency contraceptives back to a prescription requirement after a previous switch to OTC status, as in Poland.

This study contributes in two way. First, we extend the existing literature on OTC switches by examining the whole of Europe at once on the basis of a comprehensive data set. We exploited variation in the timing of OTC switches across European countries to causally analyze their effect on the use of emergency contraceptives and regular contraceptives, as well as on demographic variables and STI incidence rates using multiple staggered Difference-in-differences (DID) approaches. Second, we used two-way fixed effects DID as well as synthetic DID to analyze Poland’s unique Rx switch in the status of emergency contraceptives back to a prescription requirement and gain a deeper insight into the demographic and health implications of conservative policies regarding women’s bodily autonomy.

## Background

### Emergency contraceptives and OTC switches in Europe

**Fig. 1:**
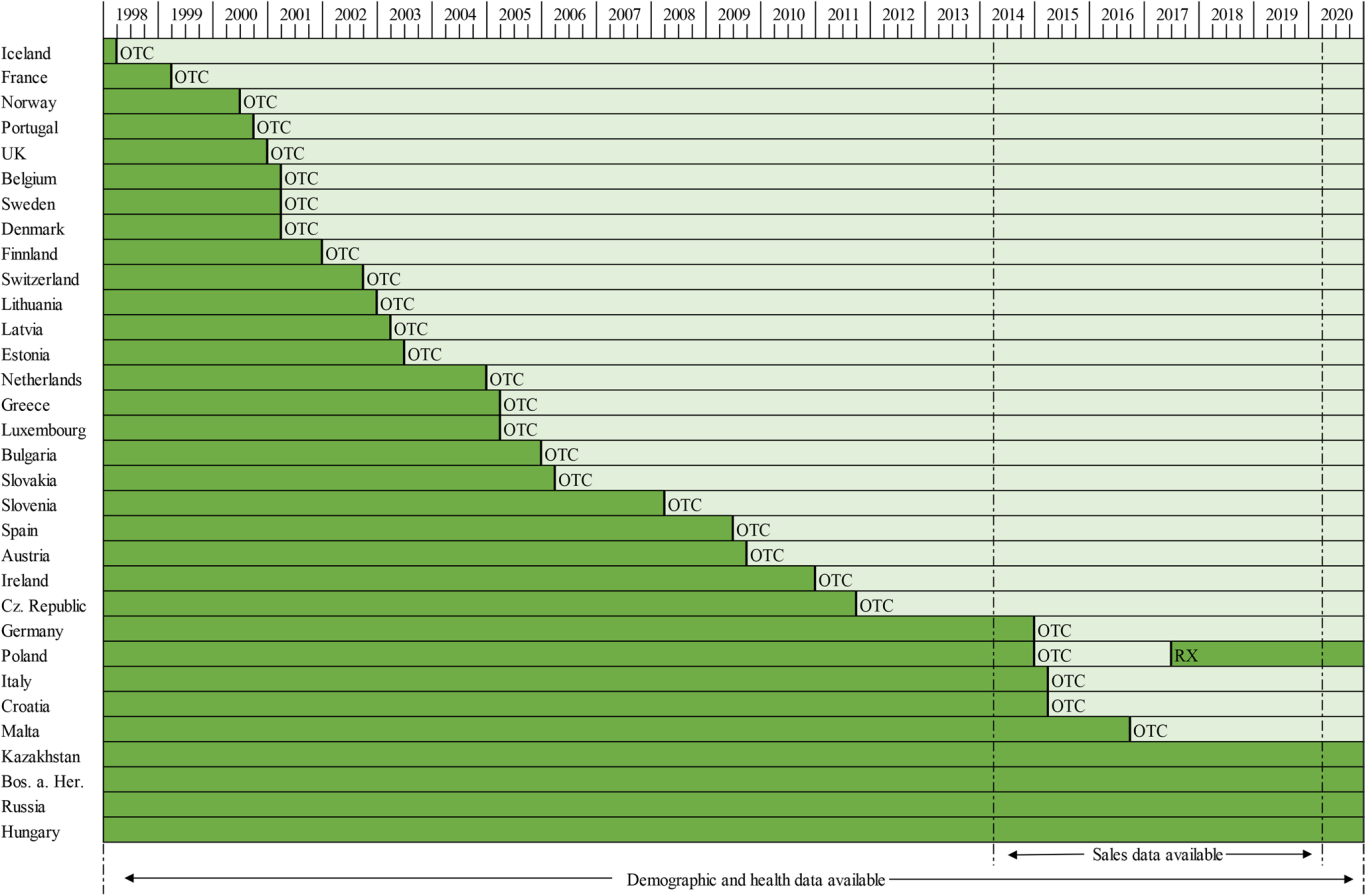
Timing of OTC and Rx switches and data availability. *Notes.* For Iceland, Denmark and Malta sales data is not available. Source: Based on multiple sources (see Table A1 in the SI). Abbreviations: OTC, over-the-counter; Rx, medical prescription; Bos. a. Her., Bosnia and Herzegovina

Levonorgestrel, introduced in 1966, is the most common emergency contraceptive in Europe. Initially a prescription pharmaceutical in nearly all EU countries, it has since been switched to over-the-counter (OTC) status in most, but not all, countries (Fig. 1 and Table A1 in the Online Supplementary Information (SI)) [6].

Ulipristal acetate, more effective than levonorgestrel, was authorized as a prescription drug by the EMA in 2009 and reclassified as over-the-counter (OTC) in 2015 [7]. This recommendation was binding for all EU/EEA members. Therefore, the countries that had not switched at least one emergency contraceptive to OTC status by then (e.g., Germany, Hungary, Italy, and Poland) were obliged to do so. Germany and Italy took this step and, at the same time, switched levonorgestrel to OTC status as well.

In July 2017, Poland reinstated the prescription requirement for ulipristal acetate, becoming the only EU country to do so, citing concerns about “harmful misuse among youths” and “early abortions” [8, 22]. Due to safety concerns, Hungary did not implement the EMA decision at all, leaving both levonorgestrel and ulipristal acetate subject to a prescription requirement [7].

Other methods of emergency contraception, such as the Yupze regimen, mifepristone, and copper IUDs, have mostly been replaced by the two emergency contraceptives described above, and their use in most European countries is negligible [23].

### Current state of literature

Some of the previous studies have examined general availability regardless of the prescription status [12, 13]. Others have assessed the prophylactic and/or free distribution of emergency contraceptives before the occurrence of potentially unprotected sexual intercourse [24–30]. However, the political intervention of an OTC switch of emergency contraceptives that we observe is different, as they still have to be acquired and paid for autonomously in a timely manner if needed. Subsequently, we focused on studies with a setting most similar to ours, i.e. a switch to prescription-free (i.e., OTC) access.

These studies have linked simplified access to emergency contraceptives to an increase in their use [6, 18, 19, 21, 31] quicker access post-intercourse [21]. Only Marston et al. [17] and Raine et al. [20] did not observe any changes in usage frequency or quantity.

Since emergency contraceptives aim to prevent pregnancies, their potential effects on birth rates and abortion rates are of high relevance. Results in the literature vary substantially by study setting. While most studies did not detect changes in the number of pregnancies or births [15, 18, 20, 21, 31, 32], the findings of Pfeifer and Stockburger [19] point to an increase in birth rates and fertility potentially due to substitution effects between emergency and regular contraceptives. Cintina and Johansen [14] noted a decrease in abortion rates, but most other studies have not found evidence of changes in this regard [15, 18, 19, 21, 31].

Given the lower effectiveness of emergency contraceptives compared to regular ones [5, 33], behavioral changes are discussed as reasons for observed variations in birth and abortion rates. Easy access to emergency contraceptives could be seen as a “last resort” option and thus induce risk-taking behavior [9, 11, 19, 34]. Atkins [9] suggested that simplified access could increase sexual activity and partnerships, though subsequent studies by the same author(s) did not confirm this [10, 11].

The use of regular birth control pills [9, 19] or condoms [11, 34] has been observed to decrease after access to emergency contraceptives was simplified. Others, however, do not support a link between easier access to emergency contraceptives and an increase in risk-taking behavior, such as unprotected sex [17, 18, 20].

Studies examining STI incidence rates as indicators of risk-taking behavior show mixed results regarding the impact of easier access to emergency contraceptives, with some not finding any effects [19, 20, 34], while others reported rising STI rates among teenagers or young adolescents [15, 16].

### Defining research gap and contribution

These heterogeneous and often contradictory results may stem from variations in study settings, such as different countries, observed population groups, and analytical methods used.

Most studies have focused only on specific outcomes relevant to our research question or on individual countries, such as France [18], the US [9–11, 14, 15, 20, 31], England respectively Great Britain [16, 17, 32] or India [34]. Only a few have examined multiple (European) countries simultaneously [6, 7, 19].

Furthermore, previous analyses have varied significantly in their populations, with some focusing on teenagers and/or young adolescents [11, 14–16, 20, 32], others on all women of reproductive age [9, 17, 18, 34] and some including broader age groups or the entire population [6, 10, 19].

Lastly, while several studies have relied on causal analysis (e.g., Difference-in-differences) [11, 14–16, 19, 20, 31, 32, 34], others have comprised literature reviews [21], correlation studies [9, 10, 17, 18] or descriptive analyses [6, 7].

Only Pfeifer and Stockburger [19] have attempted to causally analyze OTC switches and how these affect emergency contraceptive use and/or various demographic indicators (birth rates, fertility, abortion rates) and health outcomes in several European countries and the whole (fertile) population at once. Their method is somewhat similar to ours but differs in observation period, country sample, data sources, control variables, and inference statistics (see Table A2 in the SI for details). Consequently, our results and conclusions differ partially.

Hence, further causal analysis of the demographic and health impacts of OTC switches of emergency contraceptives across Europe at an aggregate level is needed to shed more light on the mixed results that have been obtained mostly from single-country studies in Europe or the USA, and to contribute a comprehensive view that helps to generalize existing results and overcome the problem of country- or outcome-specific settings.

Furthermore, there is a research gap regarding policies that restrict access to emergency contraceptives. To the best of our knowledge, no study has analyzed the unprecedented shift back to prescription-bound (Rx) status in Poland. Particularly given the emerging trends in the US and other countries to return to more restrictive abortion/contraception policies, the potential influence on demographic factors and individual contraceptive behavior are highly relevant.

## Data and methods

### Data

We approximate contraception use with quarterly sales data on emergency contraceptives (levonorgestrel and ulipristal acetate) and regular hormonal contraceptives (birth control pill, hormonal IUDs, injections, and patches) from Q2 2014 to Q1 2020 obtained from IQVIA MIDAS [35] database.^1^ IQVIA acquires data from wholesalers, retail/hospital pharmacies or manufacturers and – if necessary – extrapolates for the data to be representative for the whole country. If not stated otherwise, it includes both Rx and OTC markets. Non-hormonal methods of regular contraception (e.g., copper intrauterine devices or condoms) were excluded due to incomplete reporting for many countries and, in the case of copper intrauterine devices, we could not distinguish between regular and emergency use. Emergency contraceptive use was measured in IQVIA MIDAS standard units (SU), which reflects the number of counting units (tablets, grams, etc.) divided by the smallest common dose. In the case of regular contraceptives, the SU of regular birth control pills accounts for four weeks of effective birth control, whereas the SU of hormonal intrauterine devices accounts for the three to five protected years afforded by a single device. We addressed this by multiplying the SU of regular contraceptives by the number of quarters they effectively cover, referred to as quarterly standard units (QSU) (see Table A3 in the SI for conversion details).

With respect to potential demographic and health outcomes, the availability of contraceptives may affect birth rates, abortion rates, and the incidence rate of various sexually transmitted diseases (STIs). Data on the number of live births from 1998 to 2021 (both annually and monthly) were collected from Eurostat [36]. Data on the annual number of abortions were derived from the WHO, with gaps filled by data from national offices and Johnston’s Archive [37, 38]. As described in the background section, easier access to emergency contraceptives might impact STI incidence rates due to a change in risk-taking behavior. Yearly data on STI incidence for HIV, gonorrhoea, hepatitis B, and syphilis were obtained mainly from the European Centre for Disease Prevention and Control [39] and were complemented, whenever possible, by data from the WHO [40–43] and national statistical offices. Data missing from the aforementioned sources for Russia, Kazakhstan and Bosnia and Herzegovina were obtained from the World Bank [44]. In the case of multiple data sources, two persons individually checked consistency. To reduce potential bias from the yearly structure of the data, we considered the year after a switch to be the first year of active treatment for those countries that switched in the second half of a year (e.g., 2010 for Austria, which switched to OTC in December 2009) (see Table A5 and Table A6 in the SI for country-specific details).

We related data on sales, births, and abortions to the size of the fertile female population (i.e. women aged 15 to 49 [45–47]), data for which were derived from Eurostat [48, 49] and World Bank [44]. STI incidence rates refer to the total population (incidence per 100,000) because they directly affect both sexes (see Table A5 in the SI for country-specific details).

OTC switch dates were sourced from scientific literature or, when unavailable or conflicting, through direct inquiries with the relevant national authority (see Table A1 in the SI for country-specific details). When analyzing birth rates, we applied a one-year lag to the OTC and Rx switch dates due to the duration of pregnancy and the annual data structure.

Our analyses were based on a sample of 32 (European) countries (Austria, Belgium, Bosnia and Herzegovina, Bulgaria, Croatia, Czech Republic, Denmark, Estonia, Finland, France, Germany, Greece, Hungary, Iceland, Ireland, Italy, Kazakhstan, Latvia, Lithuania, Luxembourg, Malta, the Netherlands, Norway, Poland, Portugal, Russia, Slovakia, Slovenia, Spain, Sweden, Switzerland, and the United Kingdom).^2^

For the analysis of sales/use, data availability was limited to 2014 Q2 through 2020 Q1, during which only a few countries experienced an OTC switch: Germany, Poland, Italy, and Croatia. In the case of the Rx switch, Poland was the sole intervention country compared to 31 always- or never-treated control countries. However, for demographic and health outcomes, the data encompassed the OTC switches for all countries in the dataset. Therefore, depending on the outcome, intervention, and statistical approach, we restricted the analysis to a subset of these countries. The following chapter explains further methodological reasons along with the respective statistical methods. Table A7 in the SI offers a comprehensive overview of the country samples used in each analysis.

### Empirical strategy

#### OTC switch

We employed a staggered generalized two-way fixed effects (TWFE) difference-in-differences (DiD) approach, as follows:

1 $$\begin{aligned}\:{Y}_{\mathrm{c}\mathrm{t}}=&{{\upbeta\:}}_{0}+{{\upbeta\:}}_{1}{OTC}_{\mathrm{c}\mathrm{t}}\\&+{{\upbeta\:}}_{2,\mathrm{t}}{\:\mathrm{X}}_{\mathrm{c}\mathrm{t}}+{{\upgamma\:}}_{\mathrm{c}}+{{\uptheta\:}}_{\mathrm{t}}+{{\upepsilon\:}}_{\mathrm{c}\mathrm{t}}\end{aligned}$$

$\:{Y}_{ct}$ describes the outcome (EC/RC use, birth rate, abortion rate, and STI incidence rates). $\:{\gamma\:}_{c}\:$and $\:{\theta\:}_{t}$ constitute country- and time-fixed effects, whereas *OTC*_*ct*_ represents a dummy variable for whether country *c* facilitated OTC access to emergency contraceptives at time *t* (time is defined as quarters for use data and years for demographic and health outcomes). Thus, $\:{\beta\:}_{1}$ indicates average differences in outcomes due to an OTC switch. The vector $\:{\:X}_{ct}$ represents confounders.

To account for within-country correlated error terms in clustered data, we adopted restricted wild cluster bootstrapping with Rademacher distribution for the staggered approaches because the literature indicates that rejection rates in a case like ours with few clusters are superior to those derived from large-sample theory due to better finite-sample properties [50–53].

Importantly, incorrectly controlling for (time-varying) confounding may violate causal assumptions such as parallel trends, leading to biased average treatment effects on the treated [54, 55]. To minimize bias, we assessed potential confounders following Zeldow and Hatfield’s approach [55]. Adjusting for life expectancy, population density, or unemployment rate was not necessary because these were independent of the intervention and exhibited time-invariant marginal effects on the outcome(s). However, we included the gross domestic product (GDP) per capita, the shares of females in the total population, and a dummy variable for whether there were strict abortion laws in place, and we interacted these with time because they exhibited time-varying marginal effects on the outcome. For a full list, including our reasons for including or excluding confounders, see Table A8, Table A9, and Figure A2 in the SI. For the confounders’ data sources see Table A10 in the SI.

However, an imbalanced number of treated versus untreated countries may cause significant bias and power loss in TWFE DID analyses [56]. To mitigate this, we restricted our control countries in the TWFE DID analysis of sales/use data^3^ to “always-treated” countries that were the first to switch emergency contraceptives to OTC status (Belgium, France, Norway, Portugal, Sweden, and the UK). These countries had the highest probability that any potential long-term effects of an OTC switch had manifested by the time of analysis, minimizing their impact on the DiD results. Hungary, Russia, Kazakhstan, and Bosnia and Herzegovina were used as never-treated controls. Results for the full country sample are provided in Table A11 in the SI.

Robustness checks included several approaches. First, we added country-specific linear time trends (CSLTTs) to the TWFE DID to address potential confounding from underlying linear trends. Second, we utilized a novel estimator by Chaisemartin and D’Haultfoeuille [57, 58] to tackle the ongoing discussion in the literature about the bias of standard TWFE estimators in the presence of dynamic/heterogeneous intervention effects and staggered intervention adoption due to negative group weightings and undesired late vs. early 2 × 2 comparisons [54, 59].^4^ Finally, we employed both classic TWFE event studies in the manner of Schmidheiny and Siegloch [60] and the estimator by Chaisemartin and D’Haultfoeuille [58] to create dynamic event plots to obtain better insights into (parallel) pre-trends and treatment effect dynamics around the intervention period.

#### Rx switch

The analysis of Poland’s Rx switch differs from the previous OTC analysis due to data limitations. First, Poland is the only treated country. Second, the pre-intervention period has to be limited to the time between Poland’s OTC and Rx switch, i.e. 2015Q2 to 2017Q2 to avoid bias due to Poland’s initial OTC switch in 2015. While we had access to quarterly use/sales data and monthly birth data, other demographic and health variables were only available annually, providing just two pretreatment observations per country (see Table A4 in the SI). Lacking a single method to tackle both issues simultaneously, we used two separate approaches, each addressing one of the limitations.

First, we employed the same TWFE Difference-in-differences approach described in Eq. (1). Although this approach can handle few pre-treatment periods, it risks under-rejecting the null hypothesis when there is only one treated and many untreated clusters, even with restricted wild cluster bootstrapping or randomization inference, as previously described [56, 61]. Consequently, we employed the same reduced country sample as in the OTC switch analysis of the sales data, with the distinction that the “always-treated” and “never-treated” countries were swapped due to the contrasting intervention. (see Table A7 in the SI for details).

Second, we employed the “Synthetic Difference-in-Differences” (SDID) estimator [62], as synthetic control approaches are predestined, when there is only one treated unit. Opposed to classic synthetic control [63], which does not allow to control for unit fixed effects, and opposed to classic Difference-in-Differences, which is limited by the underlying parallel trend assumption, the SDID estimator combines both worlds by generating unit ($\:{\widehat{\omega\:}}_{c}^{sdid}$) and time ($\:{\widehat{{\uplambda\:}}}_{\mathrm{t}}^{\mathrm{s}\mathrm{d}\mathrm{i}\mathrm{d}})$ adjusted weights to build a synthetic control group with optimized parallel pretends. Hence, the algorithm for calculating the ATT (based on a two-way fixed effects regression) is as follows:

2 $$\begin{aligned}{l}\left(\widehat\tau^{sdid},\:\widehat{\mu\:},\widehat{\alpha\:},\widehat\beta\right)&=arg\;\underset{\tau,\;\mu,\;\alpha,\beta}{min}\\&\left\{\:\sum\:_{c=1}^N\sum\:_{t=1}^T(Y_{ct}-\:\mu\:-\alpha_c-\beta_t-{Rx}_{ct}\tau)^2\:\widehat{\omega\:}_c^{sdid}\widehat\lambda_t^{sdid}\right\}\end{aligned}$$

where $\:{Y}_{ct}$ represents the outcome, *Rx*_*ct*_, represents a dummy variable for whether country *c* facilitated Rx access to emergency contraceptives at time *t* (years, quarters, or months, depending on the outcome), and *α*_*c*_ and *β*_*t*_ constitute unit- and time-fixed effects. We conditioned the SDID on the same time-varying covariates as the TWFE DID before, in order to prevent bias due to potentially different evolutions of the control variables between the treatment and control groups [64].

The properties of the SDID theoretically permitted the inclusion of all countries in the Rx analysis, except for “always-treated” countries that never allowed OTC access, like Hungary. In practice, however, we had to exclude some countries due to missing values because the SDID estimator requires strictly balanced panel data^5^ (see Table A7 in the SI for details).

We address the limitations of this method due to few pre-treatment observations in the case of some demographic and health outcomes in the discussion.

## Results

### Descriptive statistics

Table 1 presents the main descriptive statistics for the outcomes and control variables. The data indicate notable differences between countries for all outcomes. For example, the use of emergency contraceptives ranged from 0.12 to 61.81 pills per quarter and 1000 women aged 15 to 49 years or the official abortion rate from 0 to 56.23 per quarter and 1000 women aged 15 to 49 years.

**Table 1:** Descriptive statistics

	*N*	Mean	Mean (pre)	Min	Max
Outcomes:					
*EC use* ^*a*^	696	16.25	5.06	0.12	61.81
*RC use* ^*b*^	696	249.68	171.44	15.03	653.37
*Birth rate* ^*c*^	768	45.25	42.10	30.92	97.70
*Abortion rate* ^*d*^	725	10.89	8.87	0	56.23
*Gonorrhoea rate* ^*e*^	670	13.33	7.60	0	121.77
*Hepatitis B rate* ^*e*^	648	6.61	5.22	0.18	82.70
*HIV rate* ^*e*^	747	7.80	5.68	0	106.19
*Syphilis rate* ^*e*^	666	9.55	6.03	0	239.13
*Control variables*					
*GDP per capita* ^*f*^	768	31141.05	22134.91	1132.13	136701.40
*Female rate* ^*g*^	768	0.51	0.51	0.48	0.54
*Abortion law* ^*h*^	768	0.11	0.28	0	1

Abbreviations: EC, emergency contraception; RC, regular contraception Number of observations: The theoretically possible maximum number of observations for sales data between 2014q2 and 2020q1 is 696 (29 × 24 quarters), as sales Data is not available for Denmark, Malte and Iceland. The theoretically possible maximum number of observations between 1998 and 2021 is 768 (32 × 24 years). Mean, minimum and maximum values are measured across all countries and observed years/quarters. Mean (pre) only includes pre-intervention periods of treated countries

^a^ Sold emergency contraceptive units per 1000 women aged 15–49; quarterly between 2014q2 and 2020q1

^b^ Months of achieved birth control by sold regular contraceptives per 1000 women aged 15–49 per quarter between 2014q2 and 2020q1

^c^ Mean birth rate per 1000 women aged 15–49 per year between 1998 and 2021

^d^ Abortions per 1000 women aged 15–49 per year between 1998 and 2021

^e^ Incidence rates per 100000 citizens; yearly data.

^f^ Gross domestic product per capita in current USD.

^g^ Share of women in the total population.

^h^ Dummy variable indicating the existence of strict abortion laws (i.e. a ban) in the respective country and year

**Fig. 2:**
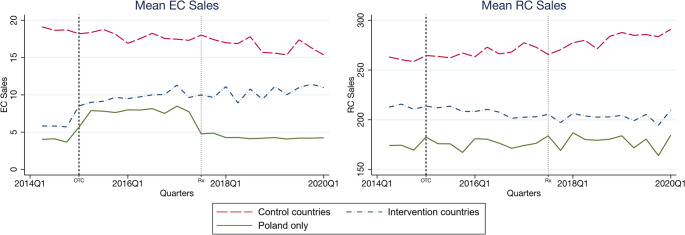
Mean sales by treatment status. *Notes.* We additionally show Poland individually, as it is the only country having switched back to Rx status. Intervention and control countries refer to the country samples defined in the according chapter and Table A7 in the SI. The vertical dashed line indicates the OTC switch, while the dotted line indicates Poland’s Rx switch. EC use in IQVIA MIDAS standard units/1000 fertile women. RC use in QSU/1000 fertile women (see methods section for QSU definition)

The descriptive data in Fig. 2 indicates a strong and sudden increase in the mean use of emergency contraceptives following the OTC switch in the intervention countries. In Poland, after the Rx switch in 2017, emergency contraceptive use declined to nearly pre-OTC switch levels. In the control countries, there was a small but steady decrease in the mean emergency contraceptives use during the post-intervention period.

The use of regular contraceptives remained mostly stable over the entire time span in the intervention countries, whereas we observed a small but steady increase in the mean use of regular contraceptives in the control countries.

More detailed descriptive statistics, broken down by country and time of intervention, can be found in Table A12 in the SI.

### OTC switch

The results of the staggered TWFE DiD model showed a significant increase in the use of emergency contraceptives following an OTC switch across all model specifications. With covariates included, the use increased by 6.062 SU per 1,000 fertile females (*p* < 0.001 for cluster robust standard errors, *p* = 0.082 for wild bootstrapped standard errors) (see Table 2). This corresponds to an increase of 119.80% compared with the mean pre-intervention use in the intervention countries. However, we did not observe any significant effect on the use of regular contraceptives following an OTC switch (0.110 < *p* < 0.286).

**Table 2:** Regression results OTC switch

	**TWFE Results**			**Robustness Check**
	(I): Basic Model	(II): + Covariates	(III): + Covariates + Wboot	dC & D’H (2020b) ^a^
Use				
Emergency contraception	6.335*** [3.931; 8.740]	6.062*** [3.993; 8.131]	6.062* [1.513; 9.389]	6.318*** [2.681; 9.956]
Regular contraception	−15.442 [−31.391; 0.507]	−15.447 [−35.265; 4.370]	−15.447 [−47.525; 6.697]	−4.245 [−16.281; 7.790]
Demographic outcomes				
Birth rate	−1.087 [−4.327; 2.154]	−0.211 [−3.089; 2.666]	−0.211 [−3.129; 2.617]	−1.936 [−4.716; 0.844]
Abortion rate	1.076 [−1.553; 3.706]	0.400 [−1.839; 2.640]	0.400 [−1.798; 2.619]	1.914 [−0.536; 4.365]
Health outcomes				
Gonorrhoea	4.626 [−5.469; 14.720]	2.617 [−6.280; 11.514]	2.617 [−6.035; 11.819]	5.350 [−1.864; 12.564]
Hepatitis B	0.416 [−2.589; 3.422]	−0.162 [−2.820; 2.496]	−0.162 [−2.883; 2.570]	1.594 [−1.515; 4.702]
HIV	−0.793 [−2.813; 1.228]	−0.967 [−2.953; 1.020]	−0.967 [−2.940; 1.000]	−1.761 [−4.066; 0.543]
Syphilis	14.176 [−2.328; 30.680]	11.886 [−2.486; 26.258]	11.886 [−2.507; 27.242]	6.315** [0.376; 12.254]

*p* < 0.1 (*), *p* < 0.05 (**), *p* < 0.01 (***). TWFE models I and II with cluster robust standard errors. 90% confidence intervals are shown in brackets. Modell II includes the GDP per capita, the share of females in the total population and abortion laws as covariates. Model III includes more conservative wild t-bootstrap standard errors (1000000 reps). Emergency contraception sales in IQVIA MIDAS standard units/1000 women aged 15–49. Regular contraception sales in months of achieved birth control by regular contraceptives per 1000 women aged 15–49. Births are measured in annually live births per 1000 fertile women. Abortions are measured in yearly abortions per 1000 fertile women. STIs are measured in incidence rate (per 100,000 population). Abbreviations: OTC, over-the-counter; TWFE, Two-way fixed effects; Wboot, Wild cluster bootstrapping; dC & D’H, de Chaisemartin & D’Haultfoeuille

^a^ ‘DID_M’ - estimator by de Chaisemartin & D’Haultfoeuille (2020a); Block bootstrapped standard errors at country level (reps: 1000). Without controls as the number of control variables exceeds the sample size in some bootstrap replications, which would return noisy estimates (especially for sales/use regressions). The table reports the difference between the average number of treatment units’ first-time switchers received after their first switch and the number of treatment units they would have received if they had never switched is used to scale the average of the periodical effects computed by the estimator by de Chaisemartin & D’Haultfoeuille (2020b). Please see their paper for more details

We also did not observe significant responses to an OTC switch for birth rate (*p* = 0.923), abortion rate (*p* = 0.816), or STI rates (0.321 < *p* < 0.924).

After incorporating country-specific linear time trends (CSLTT), the effects on EC use remained statistically significantly positive but were diminished (EC: 2.548 vs. 6.062; see Table A13 in the SI). This was expected, as linear time trends might absorb some of the post-intervention effects. The use of RC remained insignificant.

The treatment effects obtained from the alternative estimator by Chaisemartin and D’Haultfoeuille (2020b) support our findings for both emergency and regular contraceptive use following an OTC switch. For emergency contraceptive use, the effect size was slightly greater (6.318 vs. 6.062) compared to the TWFE specification that included confounders and wild cluster bootstrapping, and was also highly significant (*p* < 0.01). For regular contraceptives, the estimator did not indicate a response to the OTC switch (see Table 2). The estimator confirmed the non-significant TWFE results for all demographic indicators (birth rate, abortion rate) and STI rates, except for the syphilis rate, which showed a significant increase of 6.315 cases per 100,000 people after an OTC switch (*p* = 0.037), although all TWFE results being non-significant.

**Fig. 3:**
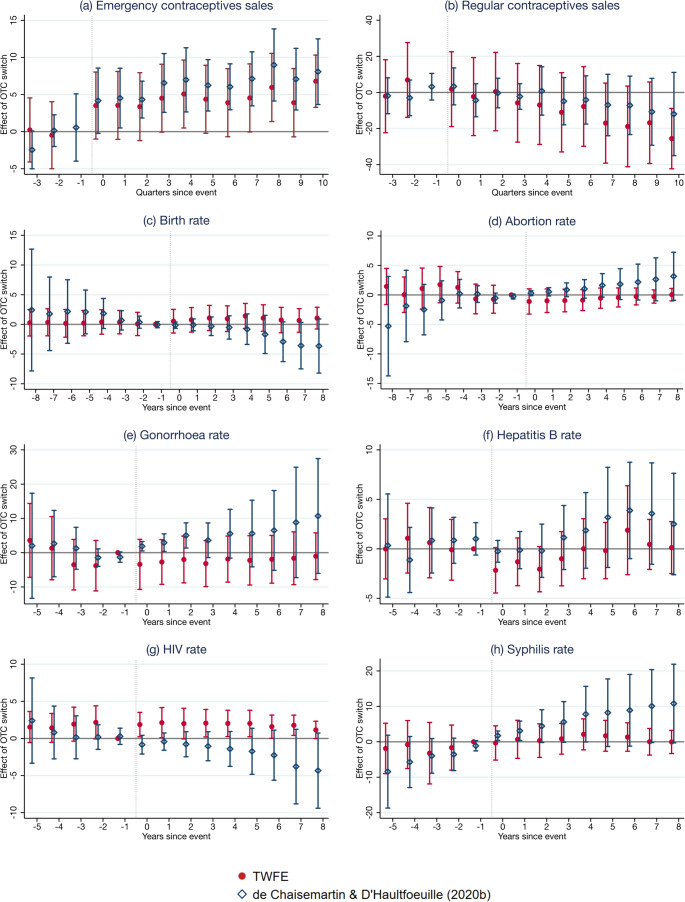
Event study plots (OTC switch). *Notes.* Plots were created with event_plot Stata module by Borusyak et al. [65]. 90% confidence intervals. de Chaisemartin & D’Haultfoeuille (2020b) estimator without control variables as otherwise the number of control variables in the event study exceeded the sample size in some bootstrap replications, which would return too noisy estimates (especially for sales/use regressions). EC use in IQVIA MIDAS standard units/1000 fertile women. RC use in QSU/1000 fertile women (see methods section for QSU definition). Births/abortion rates per 1,000 fertile women. STIs are measured in incidence rate (per 100,000 population)

Event plots showed that the effects before an OTC switch were non-significant (see Fig. 3), supporting the assumption of no (major) post-intervention difference between the intervention and control countries in the absence of a treatment (parallel trend assumption). For emergency contraceptive use after an OTC switch, we observed a significant and stable increase from the intervention year onwards (t = 0). For regular contraceptives use, birth rate, abortion rate and the gonorrhea, hepatitis B, HIV and syphilis rates, we did not observe statistically significant post-intervention trends.

### Rx switch

After emergency contraceptives were switched back to prescription status in Poland in 2017, their use decreased significantly by 3.811 SU per 1,000 fertile women (*p* = 0.047) in the SDID model. This model is preferred in the case of only one treated cluster because of the low power of wild bootstrapping under such conditions [56]. Indeed, the results of the TWFE model indicated a reduction of 3.858 SU per 1,000 fertile women for the TWFE model with covariates, which became non-significant when including wild cluster bootstrapping standard errors (see Table 3).

**Table 3:** Regression results Rx switch

	**TWFE results**			**Robustness check**
	(I): Basic Model	(II): + Covariates	(III): + Covariates + Wboot	Synth. DiD ^a^
Use				
Emergency contraception	−3.054*** [−4.642; −1.497]	−3.858*** [−4.710; −3.006]	−3.858 [−14.568; 5.927]	−3.811 [−7.577; −0.045]
Regular contraception	−5.191 [−15.167; 4.785]	12.889*** [7.335; 19.056]	12.889 [−41.697; 66.343]	5.684 [−25.594; 36.963]
Demographic outcomes				
Birth rate	−0.154 −0.236; 0.715]	−0.033 [−0.105; 0.039]	−0.033 [−0.644; 0.558]	−0.266 [−0.598; 0.066]
Abortion rate	0.718 [−0.209; 1.644]	−0.321 [−0.870; 0.227]	−0.321 [−5.518; 4.793]	0.206 [−1.673; 2.085]
Health outcomes				
Gonorrhoea	−4.364 [−10.288; 1.560]	−5.193 [−10.449; 0.062]	−5.193 [−57.180; 43.752]	−5.004 [−31.369; 21.360]
Hepatitis B	0.588 [−3.187; 4.362]	2.731** [0.889; 4.572]	2.731 [−10.060; 15.827]	−0.365 [−18.756; 18.026]
HIV	1.831 [−0.278; 3.939]	0.194 [−0.452; 0.840]	0.194 [−3.898; 4.320]	1.303 [−1.790; 4.396]
Syphilis	−0.081 [−2.120; 1.958]	1.552** [0.480; 2.624]	1.552 [−6.228; 8.372]	−0.842 [−13.206; 11.522]

*p* < 0.1 (*), *p* < 0.05 (**), *p* < 0.01 (***). 90% confidence intervals are shown in brackets. TWFE models I and II with cluster robust standard errors. Modell II includes the GDP per capita, the share of females in the total population and abortion laws as covariates. Model III includes more conservative wild t-bootstrap standard errors (1000000 reps). Emergency contraception sales in IQVIA MIDAS standard units/1000 women aged 15–49. Regular contraception sales in months of achieved birth control by regular contraceptives per 1000 women aged 15–49. Births are measured in monthly live births per 1000 fertile women. Abortions are measured in yearly abortions per 1000 fertile women. STIs are measured in incidence rate (per 100,000 population). Abbreviations: OTC, over-the-counter; TWFE, Two-way fixed effects; Wboot, Wild cluster bootstrapping; SDID, Synthetic difference-in-differences

^a^ Synthetic DiD by Arkhangelsky et al (2021). We only include time periods after Poland's initial OTC switch in order to compare only OTC periods before the Rx switch ( 2015 - 2021). The Synthetic DiD model includes the same covariates as TWFE model II and III (GDP per capita, female rate in total population, abortion laws)

**Fig. 4:**
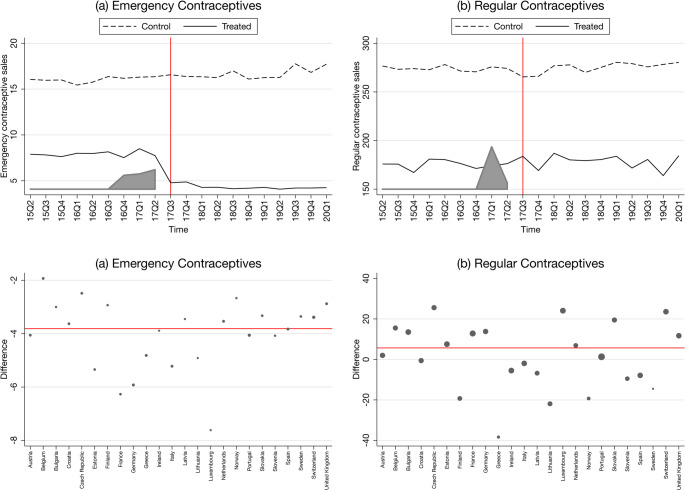
Rx switch analysis with the SDID estimator by Arkhangelsky et al. (2021). *Notes.* The upper graphs show the outcome trends before/after Poland’s Rx switch. The vertical line represents the date of Poland’s switch back to prescription bound status, i.e. the first treated point in time. The *Treated* group refers to Poland. Control refers to the weighted synthetic control group created by the SDID estimator. The lower graphs report the individual country-by-country adjusted difference in outcomes. The horizontal line represents the estimated effect (weighted average of the adjusted differences). The relative weights are indicated by the size of the markers. Emergency contraception use in IQVIA MIDAS standard units/1000 fertile women. Regular contraception use in QSU/1000 fertile women (see methods section for QSU definition). The Synthetic DiD model includes the same covariates as TWFE model II and III (GDP per capita, female rate in total population, abortion laws)

However, an effect is also clearly visible in the graphical representation of the trends of EC use in Poland compared with the synthetic control group (Fig. 4). This effect corresponds to an effect of −48.18% compared with the mean EC use in Poland between the OTC and the Rx switch (7.910 SU per 1000 fertile women). Taken together, these results suggest that the use of ECs in Poland was significantly reduced after the Rx switch.

The use of regular contraceptives showed a significant increase of 12.889 QSU per 1,000 fertile women after the Rx switch only when including covariates (Table 3). This corresponds to an increase of approx. 7.35% compared with the mean RC use (175.331 QSU per 1000 fertile women) in Poland between the OTC and Rx switches. However, when we applied more conservative wild bootstrapped standard errors, significance disappeared. Contrary to the EC sales results, we cannot assume that an underlying true effect is not found simply due to a lack of power in the regression, as both the TWFE event study (Figure A3 in the SI) and the superior^6^ SDID method (Table 3; Fig. 4) failed to provide any evidence of a significant effect regarding RC use.

For hepatitis B and syphilis rates, we observed a similar situation to that of RCs: For hepatitis B, the TWFE model including covariates indicated an increase of 2.731 cases per 100,000 people, which would reflect a change of about − 28.45% compared to the mean incidence rate (9.60 per 100,000 people) in Poland between the OTC and the Rx switch. For syphilis rate, we observed an increase of 1.552 cases per 100,000 people, which would reflect a change of about 46,61% compared to the mean incidence rate (3.330 per 100,000 people) in Poland between the OTC and the Rx switch. However, once again, neither result was confirmed by the more conservative wild bootstrapped inference statistics or SDID.^7^ For the birth rate, we observed a significant decrease (−0.154) only in the base model, but this effect diminished when we included covariates and/or wild bootstrapping or employed the estimator by Chaisemartin and D’Haultfoeuille [58]. No significant changes were found in the abortion rate or the incidence rates of gonorrhea and HIV following Poland’s Rx switch. Detailed SDID trends and weights for all (insignificant) outcomes are available in the SI (Figure A4 and A5).

## Discussion and conclusion

### Discussion

In this study of switches in the prescription status of emergency contraceptives, we sought to identify potential effects on the use of these medicines and on demographic variables and STI incidence rates. The result of our staggered TWFE DiD for the use of emergency contraceptives after an OTC switch (6.062 units per 1,000 fertile women; +119.80%) confirms previous results derived mostly from individual country analysis at an aggregate level.

Moreover, consistent with existing studies that analyzed STI rates at the level of the entire population, we found no consistent and robust signs of increased risk-taking behavior in terms of STI incidence rates after an OTC switch. Only studies that restricted their sample to adolescents or young adults found evidence of affected STI incidence rates. The same applies to the abortion rate.

Compared to the study by Pfeifer and Stockburger [19], which most closely resembles the section on OTC switches in our study (see Table A2 in the SI), we confirm their results for emergency contraceptive use, although the data used by Pfeifer and Stockburger cover an earlier observed period for contraceptive sales.

**Fig. 5:**
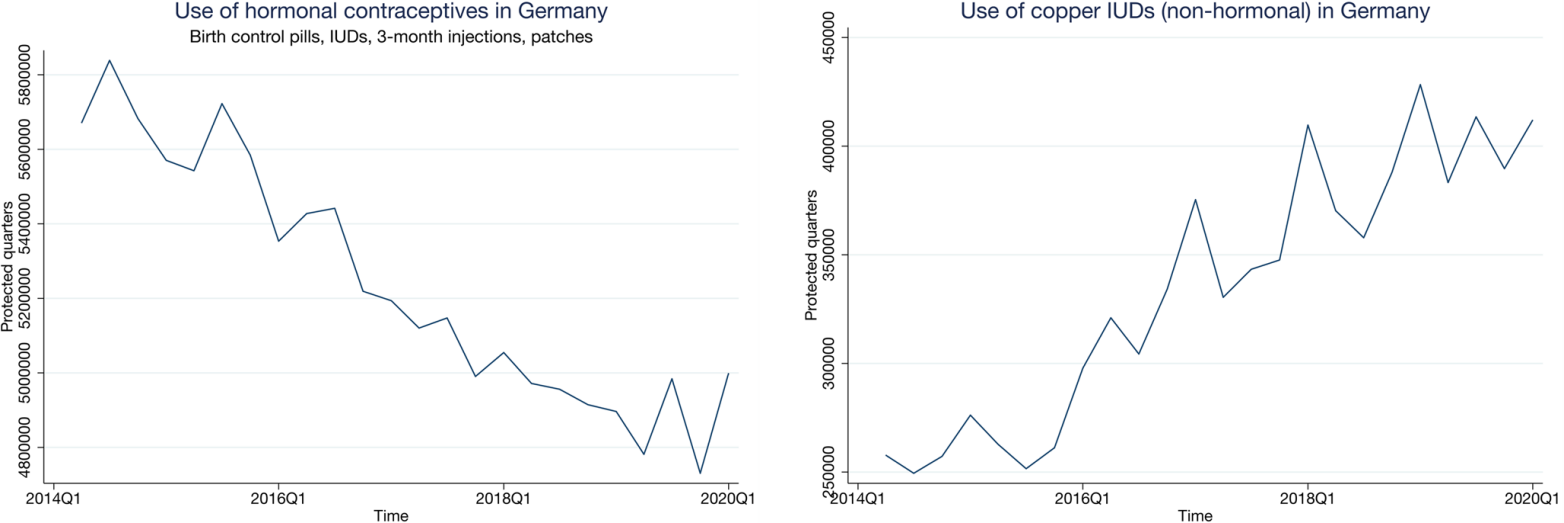
Total number of protected quarters by (non-)hormonal contraceptives in Germany. *Notes.* Protected quarters are measured in QSUs (see methods section). While the overall time protected by hormonal contraceptives decreased within the observed period, the use of the only non-hormonal contraception method that we can observe in our data, the copper IUD, increased during the same period, especially after the OTC switch in Germany

However, our results differ quite substantially for the use of regular contraceptives and birth rate. For regular contraceptives use after a switch to OTC status, our TWFE staggered approach and its robustness checks remained unremarkable. However, Pfeifer and Stockburger [19] observed a decrease in the sales of regular hormonal contraceptives in Germany after the switch to OTC status, using a Regression Discontinuity in Time design. This decrease might, at least partly, result from substitution within the class of regular contraceptives. Without adjusting IQVIA MIDAS standard units for different lengths of effectiveness, one might estimate a decrease in use. For example, the substitution of birth control pills with a three-year hormonal IUD decreases birth control pill use by about 39 standard units (one standard unit accounts for four weeks of protection with one year consisting of 52,14 weeks) but increases IUD use by only one standard unit. Thus, without adjustment (as – to the best of our knowledge - for example, in Pfeifer and Stockburger [19]), regular contraceptive use in this example decreases by 38 standard units, although only a substitution has occurred. A recent survey by the German Federal Office for Health Education suggests a trend towards decreased regular birth control pill use between 2011 and 2018, whereas the use of other forms of regular contraception (three-month injections, IUDs, vaginal rings, and condoms) remained constant and even increased in some age groups [66]. In line with this, our data also showed that in Germany, the combined use of hormonal contraceptives displayed a negative time trend from 2014 Q2 to 2020 Q1, whereas non-hormonal copper IUD use increased considerably during the same period (see Fig. 5), although this increase may only partially explain the reduced use of hormonal contraception. However, the copper IUD is the only non-hormonal contraception method that we are able to observe in our data. The OTC switch of emergency contraceptives may thus have served as an occasion for at least some individuals to reconsider hormonal contraceptives and to substitute these with non-hormonal and/or non-observed methods, such as condoms or copper IUDs. Based on these observations, we postulate that switching emergency contraceptives to OTC status does not have a substantial and generalizable negative effect on risk-taking behavior in form of the total use of regular hormonal contraceptives.

Contrary to the findings of Pfeifer and Stockburger [19] but consistent with most other European studies, our TWFE models does not indicate a positive effect on birth rate following the OTC switch of emergency contraceptives. To improve comparability with Pfeifer and Stockburger, we also conducted an analyses for the indicator fertility^8^ [67]. Analogous to our results for the birth rate, we are unable to identify any significant change in fertility as a result of an OTC switch (see Tables A14 – A20 in the SI). In addition to differences in data sources, the discrepancy between our results on birth rate and fertility and those of Pfeifer and Stockburger could also be due to differences in the time period considered, the included countries or included control variables (see Table A2 in the SI for details on differences in methodology). However, our results align with findings from the US, where OTC access did not generally impact fertility-related indicators [15, 31], except in a study by Cintina and Johansen [14] that observed fewer abortions in the 18–19 year-old subgroup.

If we assume that emergency contraceptives were used correctly by most people and are generally effective, the following two mechanisms might explain our results. First, it is possible that, in some cases, emergency contraceptives were being used immediately after purchase but that this was happening in situations in which a pregnancy was unlikely to begin with. In other words, the easier availability of emergency contraceptives might have led to more people using them in situations of very slight uncertainty, just to “be on the safe side”. This is in line with Gross et al. [31], whose theoretical model and empirical results for the US also indicate that users of EC may be rather those with lower risks of unexpected pregnancy, as uncertainty around the time of intercourse does not seem to be the primary cause of unexpected pregnancies. Second, people may have taken advantage of the easier availability of these medications and gradually stocked up on them in advance given that timely intake after unprotected intercourse is crucial to their effectiveness.

When examining the results of the switch back to prescription status in Poland in 2017, the nature of the data (only a short prescription-free period in Poland) does not allow for a perfect econometric strategy for all outcomes. For the effects on EC and RC use and birth rate, we had a sufficiently large pre-intervention period to use the SDID. However, for the only outcome with a statistically significant effect (use of EC), the direction and effect size were very similar for both models (TWFE: −3.858, SDID: −3.811) despite limitations. The decrease in the use of ECs after the Rx switch suggests that the increase in the use of emergency contraceptives observed after a switch to OTC status was actually triggered by the removal of the barrier represented by a mandatory clinical or medical consultation ahead of the medication being dispensed at pharmacies. A switch to OTC status might have decreased the risk of encountering stigma or disapproval from physicians and not being prescribed ECs due to religious beliefs or conscientious objection [68–72]. In line with this, it was shown that the use of hormonal contraception among young unmarried women in the USA correlates negatively with religious (Christian) beliefs [73]. This might be especially relevant in Poland, where the Catholic Church exerts substantial influence on reproductive health policies and attitudes about reproductive health more generally [74, 75]. With the switch back to the prescription status, these barriers were reintroduced. However, the reduction in the use of emergency contraceptives after the Rx switch was quite similar to the increase observed after the OTC switch.^9^ This suggests that changes in (perceived) moral judgment might not be permanent. When considering the other outcomes, the direction of the measured effects varied depending on the model (TWFE or SDID). The results of the SDID models are preferable for the use of RCs and birth rates because of the sufficiently large number of pre-intervention periods. For abortion rate and STI rates the respective limitations mentioned in the methodology section exist in both models.

Crucially, however, the results for all outcomes (except EC use) were not significant and, therefore, cannot be distinguished from statistical coincidence. Therefore, we feel confident enough to argue that our findings at least do not confirm the arguments put forward by the Polish government and conservative political movements in other countries to justify a switch back to prescription status and stricter reproductive health laws in general. Neither the demographic nor the health indicators after either of the switches point towards a misuse of emergency contraceptives that could be harmful or significantly impact birth or abortion rates.

Of course, one could argue that price changes after switches in prescription status drove the changes we observed in emergency contraceptive use. However, this should not substantially bias our results because the price elasticity of demand for regular contraceptives is very low (−0.04 to −0.09) [76, 77]. We are not aware of any literature on this subject specifically pertaining to emergency contraceptives, but considering the urgency of the emergency (e.g., sexual assault, unprotected intercourse, or failed protection), it seems very unlikely that price elasticity is higher for emergency contraceptives compared to regular ones.

However, our study has a number of limitations. First, we did not measure the use of all forms of contraception because our data did not cover non-pharmaceuticals. This caveat applies primarily to non-hormonal IUDs, which are classified as medical devices in most countries and hence not reported for every country, and particularly to condoms, which are widely available outside of pharmacies. Second, dispensing institutions, such as pharmacies, might influence the use of products, such as EC, independently of legal regulations by deciding not to offer them or by offering them but discouraging their use [78–80]. Third, data on abortion rates should be treated with caution when abortion laws are strict. Although official data from Poland and Ireland are very close to zero, these probably do not include a substantial number of illegal abortions or abortions performed abroad [81, 82]. Finally, cross-border shopping within the EU might also slightly bias our results on emergency contraceptive use. Citizens of countries without OTC access who live close to countries with OTC access may buy emergency contraception there.

## Conclusion

Our study adds a number of important contributions to the existing literature. First, we shed light on the topic of OTC switches of emergency contraceptives at an aggregate level that has so far been examined mainly at the national level and thus helps to generalize existing results. In this sense, we do not find sufficient evidence that, despite an increase in use, the prescription-free availability of emergency contraception significantly influences demographic or health factors on a broader scale. Second, we show for the first time, in the context of emergency contraceptives, that the effects of liberalizing regulations (i.e. an increase in the use of EC) are counteracted by returning to stricter regulations. The prescription-free availability of emergency contraceptives reduces existing barriers to acquisition, but not much of this effect remains in the event of reinstatement of prescription-only status.

## Supplementary Information

Below is the link to the electronic supplementary material.

Supplementary Material 1

## Data Availability

This article uses data from multiple sources. The pharmaceutical sales data that support the findings of this study are available from IQVIA (MIDAS). Restrictions apply to the availability of these data, which were used under license for this study and cannot be shared due to legal requirements. Further data (demographic data, birth rate, fertility, abortions, STI rates, control variables) are publicly available. Detailed information on these sources can be found in the Online Supplementary Information for each variable and country.

## Footnotes

1 For the analysis of the OTC switch, we limited the observed time span just for Poland to 2014q2–2017q2 because the switch back to Rx status in 2017Q3 constitutes a new intervention. For the analysis of the Rx switch, we only consider the time after the OTC switch 2015q1–2020q1, in order to not include a change in prescription requirements in the pre-intervention period. The same applies to demographic and health outcomes on an annual basis. See Table A4 in Appendix A for details.

2 Sales data from IQVIA are not available for a few of these countries (Denmark, Iceland, Malta, Croatia (only RC data available, See Figure A1 in Appendix A).

3 The analysis of demographic and health data is based on the full country sample (see the data section), as we can draw on a comprehensive dataset (1998–2021) that covers the OTC switches of all countries in the dataset. By contrast, sales data are limited to 2014q2–2020q1, as described in the data section.

4 The estimator by Chaisemartin & D’Haultfoeuile (DID_M) was calculated without controls as the number of control variables exceeds the sample size in some bootstrap replications, which would return noisy estimates (especially for sales/use regressions).

5 The Baltic countries were excluded from the analysis of the HIV and syphilis incidence rates due to exceptional waves of infection that peaked during the observed pre-intervention years. We also excluded Malta from the analysis of the HIV incidence rate because we observed a rising trend in the pre-intervention years unlike in the remaining sample.

6 In this case, SDID is superior to TWFE DID due to enough observed pre-intervention periods for RC use.

7 Regarding birth rate, SDID is the more appropriate method due to the sufficient number of observed pre-intervention periods. We used monthly birth rates, that is, 31 monthly observations between the OTC switch and the Rx switch.

8 Fertility refers to the average number of children a hypothetical cohort of women would have by the end of their reproductive period, as defined by the World Health Organization. Annual data on fertility from 1998 to 2021 were obtained from the United Nations’ World Population Prospects [38].See Tables A14 - A20 and Figures A6 – A7 in the Appendix for the complete analysis on fertility.

9 +119,80% EC use on average after an OTC switch and − 48.18% EC use after an Rx switch.
