# Supplementary material for: Demographic and health impacts of women’s bodily autonomy: switching prescription requirements for emergency contraceptives
Source: Eur J Health Econ. 2025 Nov 20;27(5):987–1002. doi: 10.1007/s10198-025-01863-7 (PMC13451442; doi:10.1007/s10198-025-01863-7)
Supplement: Supplementary file 1 [file 10198_2025_1863_MOESM1_ESM.docx]

**Online Supplementary Information**

**1. OTC and Rx switch dates**

| **Table A1** First-time OTC and Rx switch dates of emergency contraceptives*.* | | | | | | |  |
| --- | --- | --- | --- | --- | --- | --- | --- |
| Country | Date | Source |  | Country | Date | Source | |
| Austria | 2009q4 | Italia et al. (2020) [7]; Italia and Brand (2016) [8] |  | Kazakhstan | / |  | |
| Belgium | 2001q2 | Italia et al. (2020) [7] |  | Latvia | 2003q2 | Inquiry to State Agency of Medicines of the Republic of Latvia, answered on 22.12.2021; Pfeifer and Stockburger (2023) [20] | |
| Bosnia and Herzegovina | / |  |  | Lithuania | 2003q1 | Inquiry to State Medicines Control Agency of Lithuania, answered by Elžbieta Motūzaitė on 30.12.2021 | |
| Bulgaria | 2006q1 | Italia et al. (2020) [06]; Own inquiry to the Bulgarian Drug Agency, answered on 25.02.2021 by Lyubina Todorova, Head of Department Marketing Authorization of medicinal products |  | Luxembourg | 2005q2 | Italia and Brand (2016) [8] | |
| Croatia | 2015q2 | Italia et al. (2020) [7] |  | Malta | 2016q4 | Italia et al. (2020) [7] | |
| Czech Republic | 2011q4 | Italia et al. (2020) [7]; Pfeifer and Stockburger (2023) [20] |  | Netherlands | 2005q1 | Italia et al. (2020) [7] | |
| Denmark | 2001q2 | Italia et al. (2020) [7]; Italia and Brand (2016) [8]; Pfeifer and Stockburger (2023) [20] |  | Norway | 2000q3 | Italia et al. (2020) [7]; Grainer at al. (2003) [83] | |
| Estonia | 2003q3 | Italia et al. (2020) [7]; Pfeifer and Stockburger (2023) [20] |  | Poland ^a^ | 2015q1 (OTC) 2017q3 (Rx) | Pfeifer and Stockburger (2023) [20]; Padala et al. (2016) [84]; Italia et al. (2020) [7] | |
| Finland | 2002q1 | Italia et al. (2020) [7]; Italia and Brand (2016) [8] |  | Portugal | 2000q4 | Italia et al. (2020) [7];Grainer et al. (2003) [83]; Pfeifer and Stockburger (2023) [20] | |
| France | 1999q2 | Italia et al. (2020) [7]; Grainer at al. (2003) [83]; Italia and Brand (2016) [8]; Pfeifer and Stockburger (2023) [20] |  | Russia | / |  | |
| Germany | 2015q1 | Italia et al. (2020) [7]; Italia and Brand (2016) [8]; Pfeifer and Stockburger (2023) [20] |  | Slovakia | 2006q2 | Pfeifer and Stockburger (2023) [20] | |
| Greece | 2005q2 | Italia et al. (2020) [7]; Pfeifer and Stockburger (2023) [20] |  | Slovenia | 2008q2 | Inquiry to the Agency of the Republic of Slovenia for Medicinal Products and Medical Devices, answered by Lidija Dobrota on 02.12.2021 | |
| Hungary | / |  |  | Spain | 2009q3 | Italia et al. (2020) [7]; Pfeifer and Stockburger (2023) [20] | |
| Iceland | 1998q2 | Italia et al. (2020) [7] |  | Sweden | 2001q2 | Italia et al. (2020) [7]; Grainer at al. (2003) [83]; Pfeifer and Stockburger (2023) [20] | |
| Ireland | 2011q1 | Italia et al. (2020) [7] |  | Switzerland | 2002q4 | Italia et al. (2020) [7]; Pfeifer and Stockburger (2023) [20] | |
| Italy | 2015q2 | Italia et al. (2020) [7];[20] |  | United Kingdom | 2001q1 | Italia et al. (2020) [7]; Italia and Brand (2016) [8]; Pfeifer and Stockburger (2023) [20] | |

*Notes*:

^a^ Some sources state 2015q2 as Poland's OTC-switch. These partly refer to the law "Dz.U. 2015 poz. 477", which only restricts the minimum age of dispensing oral emergency contraception (ATC G03A) which is labelled as OTC to 15 years; see also <https://www.ginekologiaipoloznictwo.com/articles/ellaone--students-knowledge-and-opinions--part-one.pdf>

**2. Differences to Pfeifer & Stockburger (2023)**

| **Table A2** Differences in setting between our study and Pfeifer & Stockburger (2023) | | |
| --- | --- | --- |
|  | Our study | Pfeifer & Stockburger (2023) |
| Research objective | OTC switch  Rx switch | OTC switch |
| Observation period | Sales: 2014q2 – 2020q1  Health & Demography: 1998 – 2021 | Sales: 2005q1 – 2016q3  Health & Demography: 1999 – 2016 |
| Country sample | 32 countries: Austria, Belgium, Bosnia and Herzegovina, Bulgaria, Croatia, Czech Republic, Denmark, Estonia, Finland, France, Germany, Greece, Hungary, Iceland, Ireland, Italy, Kazakhstan, Latvia, Lithuania, Luxembourg, Malta, Netherlands, Norway, Poland, Portugal, Russia, Slovakia, Slovenia, Spain, Sweden, Switzerland and the United Kingdom | 28 countries: Albania, Austria, Belgium, Bosnia and Herzegovina, Croatia, Czech Republic, Denmark, Estonia, Finland, France, Germany, Greece, Hungary, Iceland, Ireland, Italy, Latvia, Lithuania, Luxembourg, Netherlands, Norway, Poland, Portugal, Slovakia, Spain, Sweden, Switzerland and the United Kingdom |
| Data sources | Sales : IQVIA MIDAS (with adjustment for duration of protection)  Health & Demography:   - Abortions: WHO, Johnston’s Archive - Births: Mostly Eurostat, partly World Bank - Population: mostly Eurostat, partly World Bank - STIs: mostly WHO and European Centre for Disease Prevention and Control | Sales : IQVIA MIDAS  Health & Demography:   - Abortion: Eurostat, The Global Life Campaign - Births: World Bank - Fertility: World Bank - Fertility by age group: Eurostat - Population: mostly Eurostat, partly World Bank - STIs: WHO |
| Control variables | GDP per capita, shares of females in the total population, strict abortion laws.  All interacted with time, as proposed by Zeldow & Hatfield (2021) | GDP per capita, unemployment rate, male and female youth rate (share of 15 to 19-year-olds), female rate, population density, life expectancy at birth, strict abortion laws.  No interactions with time |
| Statistical approach | OTC switch:   - Two-way fixed effects difference-in-differences - de Chaisemartin & D’Haultfeouille (2020b)   Rx switch:   - Two-way fixed effects difference-in-differences - Synthetic difference-in-differences | OTC switch:   - Two-way fixed effects difference-in-differences - Stacked difference-in-differences - Regression Discontinuity in Time |
| Inference statistics | Wild cluster bootstrapping (TWFE), Placebo regression (SDID), Block bootstrapping (de Chaisemartin & D’Haultfeouille, 2020b) | Randomization inference |

*Notes*: Study characteristics are extracted from Pfeifer & Stockburger (2023).

Abbreviations: SDID, Synthetic Difference-in-differences; TWFE, Two-way fixed effects; WHO, World Health Organization.

^a^ Figure and Table numbers refer to Pfeifer & Stockburger (2023).

**3. Regular contraceptives conversion**

| **Table A3** Regular contraceptives conversion | | | | |
| --- | --- | --- | --- | --- |
| Method |  | Use in IQVIA MIDAS data |  | Conversion |
|  |  |  |  |  |
| Birth Control Pill |  | Ether 21 or 28 intakes needed for four weeks of effective contraception. IQVIA SUs account for this: One SU equals four weeks. |  | QSU = SU * 0.3069 ^a b^ |
| 3-month-injection |  | One SU equals one quarter, respectively three months |  | QSU = SU * 1 |
| Hormonal IUD ^c^ |  |  |  |  |
| 13,5mg (“Jaydess”) |  | One SU equals three years |  | QSU = SU * 4 * 3 |
| 19,5mg (“Kyleena”) |  | One SU equals five years |  | QSU = SU * 4 * 5 |
| 52mg (“Mirena”) ^d^ |  | One SU equals five years |  | QSU = SU * 4 * 5 |
| Hormonal Patch |  | One SU equals one patch which lasts one week. Three weeks of intake needed for four weeks of effective contraception |  | QSU = SU / 9.77625 ^e^ |

*Abbreviations*: SU, standard unit; QSU, quarterly standard unit; IUD, intrauterine device

^a^ Potential bias: Some women continue to take the regular pill (21 intakes) without monthly interruption. The actual QSUs might therefore be slightly lower then visible in our data.

^b^ One quarter (QSU) equals 13,035 weeks (52,14/4). 1 SU = 4 weeks. 1 / 4 SUs = 1 / 13,035 QSU => 1 SU = 1 / 13,035 *4 QSU = 0,3069 QSU.

^c^ Potential bias: We cannot control for early removal (e.g. due to child wish).

^d^ Potential bias: Second approved indication of hypermenorrhea; differentiation not possible.

^e^ One quarter equals 13,035 weeks (52,14/4). 75% of weeks is intake needed: 13,035 weeks * 0,75 = 9,77625 weeks of intake per quarter. Quarterly intake: 9,77625 patches needed.

**4. Included (pre-intervention) periods**

| **Table A4** Included (pre-intervention) periods*.* | | | | | | | | | |
| --- | --- | --- | --- | --- | --- | --- | --- | --- | --- |
|  | OTC-switch | | | |  | Rx-switch | | | |
| Outcome | Total observed time span | Pre-intervention time span | Measure | Pre-periods |  | Total observed time span | Pre-intervention time span | Measure | Pre-periods |
| Sales – Emergency contraceptives | 2014q2 – 2017q2 | 2014q2 – 2014q4 | Quarters | 3 |  | 2015q1 – 2020q1 | 2015q1 – 2017q2 | Quarters | 10 |
| Sales – Regular contraceptives | 2014q2 – 2017q2 | 2014q2 – 2014q4 | Quarters | 3 |  | 2015q1 – 2020q1 | 2015q1 – 2017q2 | Quarters | 10 |
| Birth rate | 1998 – 2017 | 1998 – 2015  (lagged) | Years | 18 |  | 9.2015 – 12.2020 | 9.2015 – 4.2018  (lagged) | Monthly | 32 |
| Abortions | 1998 – 2016 | 1998 - 2014 | Years | 17 |  | 2015 – 2021 | 2015 - 2016 | Years | 2 |
| Gonorrhoea \| 2016 - 2017 \| Years \| 2 | 1998 – 2016 | 1998 - 2014 | Years | 17 |  | 2015 – 2021 | 2015 - 2016 | Years | 2 |
| Hepatitis B | 1998 – 2016 | 1998 - 2014 | Years | 17 |  | 2015 – 2021 | 2015 - 2016 | Years | 2 |
| HIV | 1998 – 2016 | 1998 - 2014 | Years | 17 |  | 2015 – 2021 | 2015 - 2016 | Years | 2 |
| Syphilis | 1998 – 2016 | 1998 - 2014 | Years | 17 |  | 2015 – 2021 | 2015 - 2016 | Years | 2 |

*Notes*. Observed periods that are included in each analysis.

**5. Data sources**

| **Table A5** Demographic data sources | | | | | | |
| --- | --- | --- | --- | --- | --- | --- |
| Country |  | Abortions ^a^ |  | Births (live) ^f^ |  | Population (annually) ^g^ |
| Austria |  | 1998-2005; 2020: Johnston's Archive ^b^ 2006-2021: not available |  | Annually: 1998-2021: Eurostat Monthly: 1998-2021: Eurostat |  | 1998-2021: Eurostat |
| Belgium |  | 1998-2021: WHO |  | Annually: 1998-2021: Eurostat Monthly: 1998-2021: Eurostat |  | 1998-2021: Eurostat |
| Bosnia and  Herzegovina |  | 2001-2016: Johnston’s Archive  2017-2019: WHO  2020-2021: Johnston’s Archive |  | Annually: 1998-2021: World Bank ^i^  Monthly: / |  | 1998-2021: World Bank ^i^ |
| Bulgaria |  | 1998-2021: WHO |  | Annually: 1998-2021: Eurostat Monthly: 1998-2021: Eurostat |  | 1998-2021: Eurostat |
| Croatia |  | 1998-2021: WHO |  | Annually: 1998-2021: Eurostat Monthly: 1998-2021: Eurostat |  | 1998-2021: Eurostat |
| Czech Republic |  | 1998-2021: WHO |  | Annually: 1998-2021: Eurostat Monthly: 1998-2021: Eurostat |  | 1998-2021: Eurostat |
| Denmark |  | 1998-2021: National Office ^c^ |  | Annually: 1998-2021: Eurostat Monthly: 1998-2021: Eurostat |  | 1998-2021: Eurostat |
| Estonia |  | 1998-2021: WHO |  | Annually: 1998-2021: Eurostat Monthly: 1998-2021: Eurostat |  | 1998-2021: Eurostat |
| Finland |  | 1998-2021: WHO |  | Annually: 1998-2021: Eurostat Monthly: 1998-2021: Eurostat |  | 1998-2021: Eurostat |
| France |  | 1998-2019: WHO 2020/21: National Office ^d j^ |  | Annually: 1998-2021: Eurostat Monthly: 1998-2021: Eurostat |  | 1998-2021: Eurostat |
| Germany |  | 1998-2021: National Office ^e^ |  | Annually: 1998-2021: Eurostat Monthly: 1998-2021: Eurostat |  | 1998-2021: Eurostat |
| Greece |  | 1998-2015: WHO 2016 - 2020: not available |  | Annually: 1998-2021: Eurostat Monthly: 1998-2021: Eurostat |  | 1998-2021: Eurostat |
| Hungary |  | 1998-2021: WHO |  | Annually: 1998-2021: Eurostat Monthly: 1998-2021: Eurostat |  | 1998-2021: Eurostat |
| Iceland |  | 1998-2018, 2020/21 WHO 2019: not available |  | Annually: 1998-2021: Eurostat Monthly: 1998-2021: Eurostat |  | 1998-2021: Eurostat |
| Ireland |  | 1998-2013: not available 2014-2021: WHO |  | Annually: 1998-2021: Eurostat Monthly: 1998-2021: Eurostat |  | 1998-2021: Eurostat |
| Italy |  | 1998-2021: WHO |  | Annually: 1998-2021: Eurostat Monthly: 1998-2021: Eurostat |  | 1998-2021: Eurostat |
| Kazakhstan |  | 1998-2021: WHO |  | Annually: 1998-2021: World Bank ^i^  Monthly: / |  | 1998-2021: World Bank ^i^ |
| Latvia |  | 1998-2021: WHO |  | Annually: 1998-2021: Eurostat Monthly: 1998-2021: Eurostat |  | 1998-2021: Eurostat |
| Lithuania |  | 1998-2021: WHO |  | Annually: 1998-2021: Eurostat Monthly: 1998-2021: Eurostat |  | 1998-2021: Eurostat |
| Luxembourg |  | 1998: not available  1999-2021: Johnston's Archive ^b^ |  | Annually: 1998-2021: Eurostat Monthly: 1998-2021: Eurostat |  | 1998-2021: Eurostat |
| Malta |  | 1998-2021: WHO |  | Annually: 1998-2021: Eurostat Monthly: 1998-2021: Eurostat |  | 1998-2021: Eurostat |
| Netherlands |  | 1998-2021: WHO |  | Annually: 1998-2021: Eurostat Monthly: 1998-2021: Eurostat |  | 1998-2021: Eurostat |
| Norway |  | 1998-2021: WHO |  | Annually: 1998-2021: Eurostat Monthly: 1998-2021: Eurostat |  | 1998-2021: Eurostat |
| Poland |  | 1998-2021: WHO |  | Annually: 1998-2021: Eurostat Monthly: 1998-2021: Eurostat |  | 1998-2021: Eurostat |
| Portugal |  | 1998-2007: Johnston's Archive ^b^ 2008-2021: WHO |  | Annually: 1998-2021: Eurostat Monthly: 1998-2021: Eurostat |  | 1998-2021: Eurostat |
| Russia |  | 1998-2021: WHO |  | Annually: 1998-2021: World Bank ^i^  Monthly: / |  | 1998-2021: World Bank ^i^ |
| Slovakia |  | 1998-2021: WHO |  | Annually: 1998-2021: Eurostat Monthly: 1998-2021: Eurostat |  | 1998-2021: Eurostat |
| Slovenia |  | 1998-2021: WHO |  | Annually: 1998-2021: Eurostat Monthly: 1998-2021: Eurostat |  | 1998-2021: Eurostat |
| Spain |  | 1998-2021: WHO |  | Annually: 1998-2021: Eurostat Monthly: 1998-2021: Eurostat |  | 1998-2021: Eurostat |
| Sweden |  | 1998-2020: WHO 2013: Johnston's Archive ^b^ |  | Annually: 1998-2021: Eurostat Monthly: 1998-2021: Eurostat |  | 1998-2021: Eurostat |
| Switzerland |  | 1998-2021: WHO |  | Annually: 1998-2021: Eurostat Monthly: 1998-2021: Eurostat |  | 1998-2021: Eurostat |
| United Kingdom |  | 1998-2021: WHO |  | Annually: 1998-2021: Eurostat Monthly: 1998-2019: Eurostat; 2020-2021 n.a. |  | 1998-2020: Eurostat 2021: Only Mid21; National Office ^h^ |

*Notes:*

^a^ WHO: https://gateway.euro.who.int/en/indicators/hfa_587-7011-number-of-abortions-all-ages/

^b^ Johnston's Archive: https://www.johnstonsarchive.net/policy/abortion/index.html

^c^ https://www.esundhed.dk/home/registre/register%20over%20legalt%20provokerede%20aborter

^d^ https://drees.solidarites-sante.gouv.fr/publications/etudes-et-resultats/interruptions-volontaires-de-grossesse-une-legere-baisse-du-taux

^e^ https://www.destatis.de/DE/Themen/Gesellschaft-Umwelt/Gesundheit/Schwangerschaftsabbrueche/_inhalt.html

^f^ Eurostat: https://ec.europa.eu/eurostat/databrowser/view/DEMO_FMONTH__custom_166307/default/table?lang=en

^g^ Eurostat: https://ec.europa.eu/eurostat/databrowser/view/demo_pjangroup/default/table?lang=de

^h^ https://www.ons.gov.uk/peoplepopulationandcommunity/populationandmigration/populationestimates/datasets/populationestimatesforuken glandandwalesscotlandandnorthernireland

^i^ World Bank: World Development Indicators. <https://databank.worldbank.org/source/world-development-indicators/preview/on> (Indicator codes: SP.POP.TOTL, SP.POP.1014.FE.5Y, SP.POP.1519.FE.5Y, SP.POP.2024.FE.5Y, SP.POP.2529.FE.5Y, SP.POP.3034.FE.5Y, SP.POP.3539.FE.5Y, SP.POP.4044.FE.5Y, SP.POP.4549.FE.5Y, SP.POP.TOTL.FE.IN, SP.DYN.TFRT.IN, SP.DYN.CBRT.IN )

^j^ https://drees.solidarites-sante.gouv.fr/publications-communique-de-presse/etudes-et-resultats/interruptions-volontaires-de-grossesse-la

| **Table A6** Health data sources | | | | | | | | |
| --- | --- | --- | --- | --- | --- | --- | --- | --- |
| Country |  | Gonorrhoea ^a b^ |  | Hepatitis B ^a c^ |  | HIV ^a d^ |  | Syphilis ^a e^ |
| Austria |  | 1998-2012: WHO 2013-2020: n.a. |  | 1998-2001,2003-2005,2010-2012: WHO 2014-2021: ECDC 2002,2006-2009,2013: n.a. |  | 1998-2019: WHO 2020,2021: ECDC |  | 1998-2012: WHO 2013-2021: n.a. |
| Belgium |  | 1998-2013: WHO 2014-2020: n.a. |  | 1998-2003,2005,2007-2008,2013: WHO 2004,2006,2009-2012,2014-2021: n.a. |  | 1998-2018: WHO 2019-2021: ECDC |  | 1998-2007, 2010, 2012-2013: WHO 2008-2009,2011,2014-2021: n.a. |
| Bosnia and  Herzegovina |  | 2002,2005-2008,2010,2011: WHO  2015-2017:Barbaric et al (2022) ^k^ |  | 1998-1999, 2002-2012: WHO  2001, 2013-2021: not available |  | 1998-2019: WHO  2020-2021: ECDC |  | 1998-2001,2003-2004,2009,2013-2015,2018-2021: n.a.  2002,2005-2008,2010-2012: WHO  2016,2017: Barbaric et al (2022) ^k^ |
| Bulgaria |  | 1998-2021: ECDC |  | 1998-2008,2010-2012: WHO 2014-2021: ECDC; 2009,2013: n.a. |  | 1998-2019: WHO 2020-2021: ECDC |  | 1998-2021: ECDC |
| Croatia |  | 1998-2013: WHO  2014-2021: ECDC |  | 1998-2011: WHO  2018-2021: ECDC  . |  | 1998-2019: WHO 2020-2021: ECDC |  | 1998-2013: WHO 2014-2021: ECDC |
| Czech Republic |  | 1998-2021: ECDC |  | 1998-2007: WHO 2007-2021: ECDC |  | 1998-2019: WHO 2020-2021: ECDC |  | 1998-2021: ECDC |
| Denmark |  | 1998-2021: ECDC |  | 2006-2021: ECDC 1998-2005: n.a. |  | 1998-2019: WHO 2020-2021: ECDC |  | 1998-2021: ECDC |
| Estonia |  | 1998-2021: ECDC |  | 1998-2013: WHO 2014-2021: ECDC |  | 1998-2019: WHO 2020-2021: ECDC |  | 1998-2021: ECDC |
| Finland |  | 1998-2011: WHO 2012-2021: ECDC |  | 1998-2005: WHO 2006-2019: ECDC; 2020: n.a. |  | 1998-2019: WHO 2020-2021: ECDC |  | 1998-1999: WHO 2000-2021: ECDC |
| France |  | n.a. |  | n.a. |  | 1998-2002: n.a.  2003-2019: WHO 2020-2021: ECDC |  | n.a. |
| Germany |  | 1998-2000,2014-2019: ECDC 2001-2013,2020-2021: n.a. |  | 1998-2013: WHO 2014-2021: ECDC |  | 1998-2019: WHO 2020-2021: ECDC |  | 1998-2000: WHO 2001-2021: ECDC |
| Greece |  | 1998-2021: ECDC |  | 1998-2004,2007-2013: WHO 2021: ECDC 2005-2006, 2014-2020: n.a. |  | 1998-2019: WHO 2020-2021: ECDC |  | 1998-2001: WHO 2014-2021: ECDC 2002-2013: n.a. |
| Hungary |  | 1998-2013: WHO 2014-2021 ECDC |  | 1998-1999,2001-2013: WHO 2012-2016: ECDC ^j^; 2017-2021: n.a. |  | 1998-2019: WHO 2020-2021: ECDC |  | 1998-2013: WHO 2014-2021: ECDC |
| Iceland |  | 1998-2021: ECDC |  | 1998-2001,2003-2005: WHO 2007-2021: ECDC; 2002,2006: n.a. |  | 1998-2019: WHO 2020-2021: ECDC |  | 1998-1999: n.a.  2000-2021: ECDC |
| Ireland |  | 1998-2021: ECDC |  | 1998-2005: WHO 2006-2021: ECDC |  | 1998-2019: WHO 2020-2021: ECDC |  | 1998: WHO 1999-2019: ECDC; 2020: n.a. |
| Italy |  | 1998-2021: ECDC |  | 1998-2006: WHO 2007-2021: ECDC |  | 1998-2003: n.a. 2004-2019: WHO 2020-2021: ECDC |  | 1998-2021: ECDC |
| Kazakhstan |  | 2001-2007: WHO  2016-2019: Barbaric et al (2022)^k^  2020-2021: n.a. |  | 1998-2005, 2007-2008, 2012-2013: WHO  2014-2021: n.a. |  | 1998-2019: WHO  2020-2021: ECDC ^l^ |  | 1998-2007: WHO  2016-2019: Barbaric et al (2022) ^k^  2020-2021: n.a.a |
| Latvia |  | 1998-2021: ECDC |  | 1998-2005: WHO 2006-2021: ECDC |  | 1998-2019: WHO 2020-2021: ECDC |  | 1998-2021: ECDC |
| Lithuania |  | 1998-2002: WHO 2003-2021: ECDC |  | 1998-2008,2010-2013: WHO 2009, 2014-2019: n.a.  2020-2021: ECDC |  | 1998-2019: WHO 2020-2021: ECDC |  | 1998-2002: WHO 2003-2021: ECDC |
| Luxembourg |  | 1998-2005: WHO 2006-2021: ECDC |  | 1998,2000-2001,2003-2005: WHO 2007-2021: ECDC; 1999, 2002,2006: n.a. |  | 1998-2019: WHO 2020-2021: ECDC |  | 1998-2005: WHO 2006-2021: ECDC |
| Malta |  | 1998-2005: WHO 2006-2021: ECDC |  | 1998-2006: WHO 2007-2021: ECDC |  | 1998-2000: n.a.  2001-2019: WHO 2020-2021: ECDC |  | 2000-2005: WHO 2006-2021: ECDC  1998-1999,2004: n.a. |
| Netherlands |  | 1998-2011: WHO 2012-2021: n.a. |  | 1998-2006: WHO 2007-2021: ECDC |  | 1998-2019: WHO 2020-2021: ECDC |  | 1998-2011: WHO 2012-2021: n.a. |
| Norway |  | 1998-2021: ECDC |  | 1998-2006: WHO 2007-2021: ECDC |  | 1998-2019: WHO 2020-2021: ECDC |  | 1998-2021: ECDC |
| Poland |  | 1998-2005: WHO 2006-2021: ECDC |  | 1998-2006: WHO 2007-2021: ECDC |  | 1998-2019: WHO 2020-2021: ECDC |  | 1998-2005: WHO 2006-2021: ECDC |
| Portugal |  | 1998-2021: ECDC |  | 1998-2006: WHO 2007-2021: ECDC |  | 1998-2019: WHO 2020-2021: ECDC |  | 1998-2021: ECDC |
| Russia |  | 1998-2013: WHO  2015,2016,2018: Barbaric et al (2022) ^k^  2017,2019-2021: National Office ^n^ |  | 1998-2009: WHO  2010-2021: National office ^m^ |  | 1998-1999: n.a.  2000-2009: National Office ^n^  2010-2019: WHO  2020-2021: ECDC ^l^ |  | 1998-2013: WHO  2015,2016,2018: Barbaric et al(2022)^k^  2017, 2019-2021: National Office ^n^  2014: n.a. |
| Slovakia |  | 1998-2005: WHO 2006-2021: ECDC |  | 1998-2005: WHO 2006-2021: ECDC |  | 1998-2019: WHO 2020-2021: ECDC |  | 1998-2005: WHO 2006-2021: ECDC |
| Slovenia |  | 1998-2005: WHO 2006-2021: ECDC |  | 1998-2005: WHO 2006-2021: ECDC |  | 1998-2019: WHO 2020-2021: ECDC |  | 1998-2005: WHO 2006-2021: ECDC |
| Spain |  | 1998-2021: ECDC |  | 1998-2013: WHO 2014-2021: n.a. |  | 1998-2002: n.a. 2003-2019: WHO 2020-2021: ECDC |  | 1998-2021: ECDC |
| Sweden |  | 1998-2021: ECDC |  | 1998-2005: WHO 2006-2021: ECDC |  | 1998-2019: WHO 2020-2021: ECDC |  | 1998-2021: ECDC |
| Switzerland |  | 1998-2011: WHO 2012-2021: National Office ^f^ |  | 1998-2011: WHO 2012-2021: National Office ^g^ |  | 1998-2019: WHO 2020-2021: ECDC |  | 1998,2006-2010: WHO 2011-2021: National Office ^h^  1999-2005: n.a. |
| United Kingdom |  | 1998-2019: ECDC 2020-2021: England only; UK Health Security Agency ^i^ |  | 2006-2019: ECDC 2020-2021: n.a. |  | 1998-2019: WHO 2020-2021: ECDC |  | 1998-2019: ECDC 2020-2021: England only; UK Health Security Agency ^i^ |

*Notes:*

^a^ ECDC: <https://atlas.ecdc.europa.eu/public/index.aspx>?

<https://www.ecdc.europa.eu/en/publications-data/monitoring/all-annual-epidemiological-reports>

https://www.ecdc.europa.eu/en/publications-data/hiv-aids-surveillance-europe-2024-2023-data

^b^ WHO: https://gateway.euro.who.int/en/indicators/hfa_322-2060-incidence-of-gonococcal-infection-per-100-000/

^c^ WHO: https://gateway.euro.who.int/en/indicators/hfa_318-2040-incidence-of-viral-hepatitis-b-per-100-000/

^d^ WHO: https://gateway.euro.who.int/en/indicators/hfa_349-2190-rate-of-new-hiv-diagnoses-per-100-000/

^e^ WHO: https://gateway.euro.who.int/en/indicators/hfa_320-2050-incidence-of-syphilis-per-100-000/

^f^ https://www.bag.admin.ch/bag/de/home/krankheiten/krankheiten-im-ueberblick/gonorrhoe.html

^g^ https://www.bag.admin.ch/bag/de/home/zahlen-und-statistiken/zahlen-zu-infektionskrankheiten.exturl.html/aHR0cHM6Ly9tZWxkZXN5c3RlbWUuYmFnYXBwcy5jaC9pbmZyZX/BvcnRpbmcvZGF0ZW5kZXRhaWxzL2QvaGVwX2JfdG90YWwuaHRt/bD93ZWJncmFiPWlnbm9yZQ==.html

^h^ https://www.bag.admin.ch/bag/de/home/krankheiten/krankheiten-im-ueberblick/syphilis.html

^i^ https://www.gov.uk/government/statistics/sexually-transmitted-infections-stis-annual-data-tables

^j^ <https://www.ecdc.europa.eu/sites/default/files/documents/AER_for_2016-hepatitis-B_0.PDF>

^k^ Barbaraic et al. (2022). Surveillance and epidemiology of syphilis, gonorrhoea and chlamydia in the non-European Union countries of the World Health Organization European Region, 2015 to 2020. Eurosurveillance 27(8).

^l^ <https://www.ecdc.europa.eu/sites/default/files/documents/2022-Annual_HIV_Report_final.pdf>

^m^ Rosstat indicator 3.3.4: Hepatitis B incidence per 100.000 population ( <https://eng.rosstat.gov.ru/3.3.4> )

^n^ Russian Statistical Yearbooks ( <https://eng.rosstat.gov.ru/Publications/document/74811> )

**6. EC Sales in Croatia**

**Figure A1** Emergency contraceptives sales in Croatia.

*Notes*. Sold units in absolute numbers. We had to exclude Croatia from all sales analysis as we observed a severe break in the data collection by IQVIA. Right after the OTC switch of Ulipristal Acetate (EllaOne), sales increased as excpected. However, from 2015q4 onwards, observed sales drop to a flat zero. As Ulipristal Acetate is still available in Croatia, we suspect invalid data and hence do not include it in the analysis of EC sales/use. As there are only few treated countries within our observed time span for sales data (2014q2 – 2020q1), including it would lead to severe bias

**7. Country samples**

| **Table A7** Country selection depending on intervention and outcome. | |
| --- | --- |
| Outcome | Countries |
| OTC |  |
| Sales – Emergency contraceptives | Intervention countries: Germany, Italy, Poland  Control countries: Hungary, Bosnia and Herzegovina, Russia, Kazakhstan, France, Norway, Portugal, United Kingdom, Belgium, Sweden ^a^ |
| Sales – Regular contraceptives | Intervention countries: Germany, Italy, Poland, Croatia  Control countries: Hungary, Bosnia and Herzegovina, Russia, Kazakhstan, France, Norway, Portugal, United Kingdom, Belgium, Sweden ^b^ |
| Demographic and health outcomes | Full sample (all 32 countries)  Intervention countries: Germany, Italy, Poland, Croatia |
| Rx (TWFE DID) |  |
| Sales – Emergency contraceptives | Intervention countries: Poland  Control countries: Germany , Italy, Hungary, Bosnia and Herzegovina, Russia, Kazakhstan, France, Norway, Portugal, United Kingdom, Belgium, Sweden ^a^ |
| Sales – Regular contraceptives | Intervention countries: Poland  Control countries: Germany, Italy, Croatia, Hungary, Bosnia and Herzegovina, Russia, Kazakhstan, France, Norway, Portugal, United Kingdom, Belgium, Sweden ^b^ |
| Demographic and health outcomes | Intervention countries: Poland  Control countries: Germany, Italy, Croatia, Hungary, Bosnia and Herzegovina, Russia, Kazakhstan, France, Norway, Portugal, United Kingdom, Belgium, Sweden ^b^ |
| Rx (SDID) |  |
| Sales – Emergency contraceptives | Full sample except always-treated countries (Hungary, Russia, Kazakhstan, Bosnia and Herzegovina)  Intervention countries: Poland |
| Sales – Regular contraceptives | Full sample except always-treated countries (Hungary, Russia, Kazakhstan, Bosnia and Herzegovina)  Intervention countries: Poland |
| Birth rate | Full sample except always-treated countries (Hungary, Russia, Kazakhstan, Bosnia and Herzegovina)  Intervention countries: Poland |
| Abortion rate | Full sample except always-treated countries (Hungary, Russia, Kazakhstan, Bosnia and Herzegovina) and countries with missing values (Austria, Belgium, Denmark, Greece, Iceland, Ireland, Sweden)  Intervention countries: Poland |
| Gonorrhoea | Full sample except always-treated countries (Hungary, Russia, Kazakhstan, Bosnia and Herzegovina) and countries with missing values (Austria, Belgium, France, Germany, Greece, Netherlands, Portugal)  Intervention countries: Poland |
| Hepatitis B | Full sample except always-treated countries (Hungary, Russia, Kazakhstan, Bosnia and Herzegovina) and countries with missing values (Croatia, Austria, Belgium, Bulgaria, Czech Republic, France, Greece, Lithuania, Spain, United Kingdom)  Intervention countries: Poland |
| HIV | Full sample except always-treated countries (Hungary, Russia, Kazakhstan, Bosnia and Herzegovina) and countries with missing values (Portugal)  Intervention countries: Poland |
| Syphilis | Full sample except always-treated countries (Hungary, Russia, Kazakhstan, Bosnia and Herzegovina) and countries with missing values (Austria, Belgium, France, Greece, Lithuania, Netherlands)  Intervention countries: Poland |

*Notes*: Synthetic DID does not support unbalanced data (i.e. missing values) or always treated countries. In case of Poland’s Rx switch, countries that have never facilitated OTC access to emergency contraceptives before are considered as ‘always-treated countries’. Abbreviations: OTC, over-the-counter; TWFE, Two-way fixed effects; SDID, Synthetic difference-in-differences.

^a^ All treated countries within the observed time span (except Croatia due to missing data), all never-treated countries, oldest always-treated countries.

^b^ All treated countries within the observed time span, all never-treated countries, oldest always-treated countries. Birth rate without Bosnia and Herzegovina, Kazakhstan and Russia due to non-availability of monthly data.

**8. Potential confounder assessment**

In order to assess, whether adjustment for potential confounders in the TWFE staggered DiD model is necessary, we followed the approach by Zeldow and Hatfield (2021). We assessed whether a) a confounder’s value varied in time, b) this variation is related to the intervention and c) the marginal effect on the outcome changes over time.

a) Time-variation:

The values of all considered confounders change over time on country level.

b) Relation between the intervention and variation in time:

We examined whether the intervention might influence the value of the confounder or, more generally speaking, if the confounder develops differently between control and intervention group. However, because all countries (except Hungary) were subject to the intervention at some point in time, it was not feasible to contrast trends properly between control and intervention groups in our staggered setting. We therefore employed two strategies to assess whether the intervention might affect the confounder: (A) We plotted the confounders across all countries in relation to the respective intervention time as an average across all treated countries (see Figure A2). In doing so, we sought to determine whether there might be noteworthy changes around the time of the intervention. (B) We ran the basic TWFE staggered DiD regression with the respective confounder as dependent variable (see Table A9). If both strategies were inconspicuous, we considered the confounder to be independent of the intervention (see Table A8).

| **Table A8** Potential confounders' attributes*.* | | | | | | | |
| --- | --- | --- | --- | --- | --- | --- | --- |
| Outcome | Variation in time |  | Relation of variation in time to intervention |  | Marginal effect on the outcome |  | Adjustment |
| Outcome: EC/RC sales |  |  |  |  |  |  |  |
| GDP per Capita | Time-varying |  | Independent of intervention |  | Time-varying |  | X*time |
| Life expectancy | Time-varying |  | Independent of intervention |  | Time-invariant |  | None |
| Population density | Time-varying |  | Independent of intervention |  | Time-invariant |  | None |
| Unemployment rate | Time-varying |  | Independent of intervention |  | Time-invariant |  | None |
| Women share (population) | Time-varying |  | Independent of intervention |  | Time-varying |  | X*time |
| Abortion law | Time-varying |  | Independent of intervention |  | Time-varying |  | X*time |
| Outcome: Birth rate |  |  |  |  |  |  |  |
| GDP per Capita | Time-varying |  | Independent of intervention |  | Time-varying |  | X*time |
| Life expectancy | Time-varying |  | Independent of intervention |  | Time-invariant |  | None |
| Population density | Time-varying |  | Independent of intervention |  | Time-invariant |  | None |
| Unemployment rate | Time-varying |  | Independent of intervention |  | Time-invariant |  | None |
| Women share (population) | Time-varying |  | Independent of intervention |  | Time-varying |  | X*time |
| Abortion law | Time-varying |  | Independent of intervention |  | Time-varying |  | X*time |
| Outcome: Fertility |  |  |  |  |  |  |  |
| GDP per Capita | Time-varying |  | Independent of intervention |  | Time-varying |  | X*time |
| Life expectancy | Time-varying |  | Independent of intervention |  | Time-invariant |  | None |
| Population density | Time-varying |  | Independent of intervention |  | Time-invariant |  | None |
| Unemployment rate | Time-varying |  | Independent of intervention |  | Time-invariant |  | None |
| Women share (population) | Time-varying |  | Independent of intervention |  | Time-varying |  | X*time |
| Abortion law | Time-varying |  | Independent of intervention |  | Time-varying |  | X*time |
| Outcome: Abortion rate |  |  |  |  |  |  |  |
| GDP per Capita | Time-varying |  | Independent of intervention |  | Time-varying |  | X*time |
| Life expectancy | Time-varying |  | Independent of intervention |  | Time-invariant |  | None |
| Population density | Time-varying |  | Independent of intervention |  | Time-invariant |  | None |
| Unemployment rate | Time-varying |  | Independent of intervention |  | Time-invariant |  | None |
| Women share (population) | Time-varying |  | Independent of intervention |  | Time-varying |  | X*time |
| Abortion law | Time-varying |  | Independent of intervention |  | Time-varying |  | X*time |
| Outcome: STI rates |  |  |  |  |  |  |  |
| GDP per Capita | Time-varying |  | Independent of intervention |  | Time-varying |  | X*time |
| Life expectancy | Time-varying |  | Independent of intervention |  | Time-invariant |  | None |
| Population density | Time-varying |  | Independent of intervention |  | Time-invariant |  | None |
| Unemployment rate | Time-varying |  | Independent of intervention |  | Time-invariant |  | None |
| Women share (population) | Time-varying |  | Independent of intervention |  | Time-varying |  | X*time |
| Abortion law | Time-varying |  | Independent of intervention |  | Time-varying |  | X*time |

*Notes*: Based on the framework by Zeldow and Hatfield (2021).

**Fig. A2** Time trends of potential confounders

| **Table A9** Potential confounder regression | | |
| --- | --- | --- |
| Confounders |  | Results |
| GDP per capita | Coef. | 2607.669 |
|  | p>\|t\| | 0.227 |
|  | [90%] | [-776.456; 6121.968] |
| Life expectancy | Coef. | -0.142 |
|  | p>\|t\| | 0.479 |
|  | [90%] | [-0.475; 0.192] |
| Population density | Coef. | 4.437 |
|  | p>\|t\| | 0.142 |
|  | [90%] | [-0.413; 9.665] |
| Unemployment rate | Coef. | 0.469 |
|  | p>\|t\| | 0.218 |
|  | [90%] | [-0.032; 1.019] |
| Female rate | Coef. | -0.000 |
|  | p>\|t\| | 0.571 |
|  | [90%] | [-0.001; 0.001] |
| Abortion law | Coef. | -0.064 |
|  | p>\|t\| | 0.199 |
|  | [90%] | [-0.155; 0.003] |

*Notes*: p<0.1 (*), p<0.05 (**), p<0.01 (***); TWFE DiD as in equation (2) with individual confounders as outcome (Y_ct_) instead of a confounder vector (X_ct_). Sample: Austria, Belgium, Bulgaria, Czech Republic, Denmark, Estonia, Finland, France, Germany, Greece, Iceland, Ireland, Italy, Latvia, Lithuania, Luxembourg, Malta, Netherlands, Norway, Poland, Portugal, Slovakia, Slovenia, Spain, Sweden, Switzerland, United Kingdom.

c) Change in the marginal effect of confounder on outcome:

We examined whether the marginal effect (i.e., the regression coefficient) of a confounder changes over time. We assumed that the following confounders may have had a varying marginal effect on the respective outcome:

- Contraception use/birth rate/fertility/abortions:
  - GDP per capita: Due to inflation
  - Female rate: The marginal effect might change if the OTC switch leads to an increase in the use of emergency contraceptives per woman, which, in turn, might influence use/birth/abortion rates due to the intended effect of emergency contraceptives. Therefore, a one percentage point increase in the female rate might have time-varying effect on the outcome.
  - Abortion law: The marginal effect might change because the existence of strict abortion laws could affect the extent to which emergency contraception is seen as a “last resort” solution. In this way, strict laws might amplify the effect of the OTC switch on contraceptive use/births/fertility/abortions
- STI rates:
  - GDP per Capita: Due to inflation
  - Female rate: An OTC switch could conceivably lead to decrease in the use of general contraception (for example, because it is known that other last-resort solutions exist), in turn leading to an increased risk of infection with an STI and a change in the marginal effect; the same holds for men, who might refrain from using condoms.
  - Abortion law: The existence of strict abortion laws in combination with a change in OTC status back to Rx, further reduces other potential “last resort” methods, increasing the general awareness of contraception, which in turn could lower the STI infection risk.

| **Table A10** Potential confounder data sources | | |
| --- | --- | --- |
| Potential confounder |  | Source |
| Gross domestic product per capita (current prices; USD) |  | International Monetary Fund - World Economic Outlook Database <https://www.imf.org/en/Publications/WEO/weo-database/2021/October/download-entire-database>  Last visit: 11.03.2022 |
| Female population rate |  | World Bank - Population, female (% of total population) https://data.worldbank.org/indicator/SP.POP.TOTL.FE.ZS Last visit: 16.04.2021 |
| Strict abortion law |  | Own research |
| Life expectancy |  | World Bank - Live expectancy at birth, total (years) https://data.worldbank.org/indicator/SP.DYN.LE00.IN Last visit: 11.03.2022 |
| Population density |  | Eurostat - Population density by NUTS 3 region https://ec.europa.eu/eurostat/de/web/products-datasets/product?code=demo_r_d3dens  Last visit: 11.03.2022 |
| Unemployment rate |  | European Health Information Gateway (WHO) - Indicator: "Unemployment rate", <https://gateway.euro.who.int/en/hfa-explorer/#QEQVEFoZS2>  Last visit: 10.02.2022; Schweizer Bundesamt für Statistik - "Arbeitslosigkeit, nach Agglomerationen"  Bosnia & Herzegovina: World Bank, Unemployment, total (%) (SL.UEM.TOTL.ZS) |

**8. Regression results OTC switch (sales data, full country sample)**

| **Table A11** Regression results OTC switch (sales data, full country sample) | | | | | | | | | | | |
| --- | --- | --- | --- | --- | --- | --- | --- | --- | --- | --- | --- |
|  | TWFE Results | | | | | | | |  | Robustness Check | |
|  | (I):  Basic Model | |  | (II):  + Covariates | |  | (III):  + Covariates + Wboot | |  | dC & D'H (2020b) ^a^ |  |
| Use |  |  |  |  |  |  |  |  |  |  |  |
| Emergency contraception | **5.417** | ******* |  | **5.262** | ******* |  | **5.262** | ***** |  | **6.318** | ******* |
|  | [3.839; 6.995] |  |  | [3.884; 6.641] |  |  | [2.984; 7.470] |  |  | [2.705; 9.931] |  |
| Regular contraception | -8.430 |  |  | -10.268 |  |  | -10.268 |  |  | -4.245 |  |
|  | [-23.235; 6.376] |  |  | [-24.553; 4.017] |  |  | [-30.518; 5.830] |  |  | [-16.006; 7.516] |  |

*Notes*: p<0.1 (*), p<0.05 (**), p<0.01 (***). 90% confidence intervals are shown in brackets. Modell II includes the GDP per capita, the share of females in the total population and abortion laws as covariates. EC use in IQVIA MIDAS standard units / 1000 fertile women. RC use in QSU / 1000 fertile women. Compared to the main analysis, this model includes the full country sample:. Abbreviations: OTC, over-the-counter; dC & D'H, de Chaisemartin & D'Haultfoeuille (2020b); TWFE, Two-way fixed effects. Observed time: 2015Q3 - 2020Q1

^a^ 'DID_M' - estimator by de Chaisemartin & D'Haultfoeuille (2020a); Block bootstrapped standard errors at country level (reps: 1000). Without controls as the number of control variables exceeds the sample size in some bootstrap replications, which would return noisy estimates (especially for sales/use regressions). The table reports the difference between the average number of treatment units’ first-time switchers received after their first switch and the number of treatment units they would have received if they had never switched is used to scale the average of the periodical effects computed by the estimator by de Chaisemartin & D'Haultfoeuille (2020b). Please see their paper for more details.

| **9. Descriptive statistics**  **Table A12** Descriptive statistics | | | | | | | | | | | | | | | | | | | | | | | | | |
| --- | --- | --- | --- | --- | --- | --- | --- | --- | --- | --- | --- | --- | --- | --- | --- | --- | --- | --- | --- | --- | --- | --- | --- | --- | --- |
|  |  |  | *EC use ^a^* | |  | *RC use ^b^* | |  | *Birth rate ^c^* | |  | *Abortion rate ^c^* | |  | *Gonorrhoea rate ^d^* | |  | *Hepatitis B rate ^d^* | |  | *HIV rate ^d^* | |  | *Syphilis rate ^d^* | |
|  | *Switch* |  | 2014Q2 | 2016Q1 |  | 2014Q2 | 2016Q1 |  | t-2 | t+2 |  | t-2 | t+2 |  | t-2 | t+2 |  | t-2 | t+2 |  | t-2 | t+2 |  | t-2 | t+2 |
| Austria | 2010 |  | 21.06 | 20.02 |  | 238.27 | 189.52 |  | 38.02 | 39.18 |  | / | / |  | 9.87 | 16.39 |  | / | 1.08 |  | 4.52 | 4.33 |  | 6.62 | 5.86 |
| Belgium | 2001 |  | 20.91 | 18.88 |  | 560.05 | 535.73 |  | 46.13 | 46.20 |  | 5.15 | 6.32 |  | 1.74 | 2.93 |  | 1.23 | 7.02 |  | 7.82 | 10.16 |  | 0.26 | 2.89 |
| Bulgaria | 2006 |  | 11.26 | 11.26 |  | 53.89 | 65.59 |  | 37.74 | 44.25 |  | 25.50 | 20.84 |  | 3.03 | 2.37 |  | 12.45 | 8.19 |  | 0.64 | 1.61 |  | 11.12 | 5.57 |
| Croatia | 2015 |  | 1.68 | 0.44 |  | 67.26 | 65.02 |  | 42.05 | 41.35 |  | 3.33 | 2.73 |  | 0.33 | 0.74 |  | 3.20 | 2.38 |  | 2.00 | 2.57 |  | 1.81 | 0.85 |
| Czech Republic | 2012 |  | 11.57 | 12.47 |  | 314.17 | 293.12 |  | 46.50 | 44.81 |  | 9.53 | 8.94 |  | 7.16 | 13.26 |  | 2.33 | 0.99 |  | 1.71 | 2.20 |  | 4.42 | 3.89 |
| Denmark | 2001 |  | / | / |  | / | / |  | 52.30 | 51.91 |  | 12.82 | 12.65 |  | 6.29 | 3.45 |  | / | / |  | 5.40 | 5.01 |  | 0.36 | 1.56 |
| Estonia | 2004 |  | 23.44 | 20.62 |  | 191.31 | 196.41590.68 |  | 37.77 | 44.33 |  | 31.49 | 27.99 |  | 38.89 | 20.73 |  | 17.69 | 3.34 |  | 65.18 | 49.60 |  | 20.67 | 9.25 |
| Finland | 2002 |  | 21.90 | 19.74 |  | 362.83 | 369.13 |  | 46.37 | 48.60 |  | 8.93 | 9.33 |  | 5.24 | 4.73 |  | 4.62 | 1.09 |  | 2.69 | 2.37 |  | 3.85 | 2.07 |
| France ^f^ | 1999 |  | 27.07 | 26.40 |  | 344.74 | 364.24 |  | 51.22 | 53.92 |  | 13.89 | 14.40 |  | / | / |  | / | / |  | / | / |  | / | / |
| Germany | 2015 |  | 6.96 | 11.79 |  | 322.65 | 307.03 |  | 38.36 | 45.42 |  | 5.78 | 5.86 |  | / | 21.20 |  | 0.80 | 4.40 |  | 4.01 | 3.91 |  | 6.61 | 9.12 |
| Greece | 2005 |  | 15.08 | 16.74 |  | 215.23 | 263.57 |  | 38.99 | 41.83 |  | 5.89 | 6.08 |  | 1.09 | 1.82 |  | 1.31 | 0.76 |  | 3.87 | 4.98 |  | / | / |
| Iceland ^g^ | 1998 |  | / | / |  | / | / |  | 59.91 | 60.58 |  | 13.11 | 13.86 |  | 2.20 | 3.58 |  | 5.48 | 17.43 |  | 2.92 | 3.56 |  | / | 3.23 |
| Ireland | 2011 |  | 25.47 | 26.23 |  | 311.05 | 306.48 |  | 63.34 | 59.57 |  | / | / |  | 9.62 | 27.64 |  | 17.90 | 9.30 |  | 8.86 | 7.47 |  | 2.34 | 3.54 |
| Italy | 2015 |  | 6.48 | 8.73 |  | 141.28 | 137.06 |  | 38.26 | 35.39 |  | 7.47 | 6.22 |  | 0.89 | 1.42 |  | 0.90 | 0.70 |  | 6.38 | 5.91 |  | 1.96 | 2.69 |
| Latvia | 2003 |  | 24.90 | 18.04 |  | 126.27 | 120.31 |  | 33.46 | 38.06 |  | 26.54 | 22.24 |  | 23.41 | 30.85 |  | 35.90 | 7.59 |  | 34.53 | 13.36 |  | 25.03 | 19.56 |
| Lithuania | 2003 |  | 26.21 | 24.21 |  | 95.42 | 96.92 |  | 35.16 | 34.40 |  | 15.42 | 11.63 |  | 21.15 | 12.91 |  | 11.01 | 4.24 |  | 2.07 | 3.61 |  | 25.38 | 8.79 |
| Luxembourg | 2005 |  | 20.07 | 20.62 |  | 444.78 | 387.81 |  | 47.09 | 46.09 |  | 1.68 | 1.24 |  | 1.11 | 0.21 |  | 0.22 | 2.90 |  | 11.96 | 10.21 |  | 3.32 | 2.94 |
| Malta | 2017 |  | / | / |  | / | / |  | 43.23 | 37.89 |  | 0 | 0 |  | 15.01 | 32.62 |  | 4.10 | 4.70 |  | 14.57 | 17.09 |  | 9.32 | 19.25 |
| Netherlands | 2005 |  | 0.76 | 0.58 |  | 613.34 | 633.55 |  | 50.63 | 46.55 |  | 7.29 | 7.27 |  | 9.26 | 11.15 |  | 2.02 | 1.70 |  | 6.74 | 7.69 |  | 4.42 | 3.98 |
| Norway | 2000 |  | 28.01 | 23.15 |  | 399.81 | 394.43 |  | 55.63 | 52.64 |  | 13.40 | 12.95 |  | 4.27 | 5.29 |  | 10.58 | 4.38 |  | 3.29 | 5.21 |  | 1.19 | 1.12 |
| Poland (OTC) | 2015 |  | 4.03 | 7.99 |  | 174.09 | 180.90 |  | 40.16 | 44.90 |  | 0.08 | 0.12 |  | 1.44 | 0.36 |  | 4.00 | 8.90 |  | 2.89 | 3.69 |  | 3.48 | 4.20 |
| Poland (RX) | 2017 |  | 7.52 | 4.12 |  | 171.30 | 179.31 |  | 40.73 | 42.44 |  | 0.11 | 0.12 |  | 1.32 | 0.74 |  | 9.20 | 7.50 |  | 3.32 | 3.80 |  | 3.26 | 4.28 |
| Portugal | 2001 |  | 17.03 | 17.80 |  | 387.18 | 392.23 |  | 44.65 | 42.90 |  | 0.19 | 0.27 |  | 0.26 | 0.37 |  | 3.98 | 1.13 |  | 32.36 | 23.47 |  | 1.24 | 1.33 |
| Slovakia | 2006 |  | 9.71 | 9.55 |  | 140.73 | 127.12 |  | 37.43 | 40.82 |  | 10.66 | 9.53 |  | 2.38 | 2.83 |  | 2.06 | 3.40 |  | 0.28 | 0.98 |  | 3.79 | 4.24 |
| Slovenia | 2008 |  | 14.55 | 12.09 |  | 307.27 | 261.92 |  | 37.95 | 46.30 |  | 11.29 | 8.97 |  | 1.70 | 2.15 |  | 2.80 | 2.10 |  | 1.64 | 1.71 |  | 0.80 | 1.95 |
| Spain | 2010 |  | 16.80 | 16.07 |  | 147.36 | 150.71 |  | 44.81 | 39.91 |  | 10.01 | 9.89 |  | 4.15 | 6.50 |  | 2.21 | 1.29 |  | 7.68 | 8.43 |  | 5.57 | 7.78 |
| Sweden | 2001 |  | 27.36 | 28.76 |  | 330.51 | 365.13 |  | 44.26 | 49.76 |  | 15.42 | 17.30 |  | 4.79 | 6.51 |  | 2.42 | 4.21 |  | 2.45 | 4.13 |  | 0.45 | 2.08 |
| Switzerland | 2003 |  | 10.60 | 17.24 |  | 345.71 | 327.88 |  | 40.84 | 40.04 |  | 7.02 | 5.94 |  | 6.32 | 9.17 |  | 1.69 | 1.00 |  | 8.73 | 9.72 |  | / | / |
| United Kingdom | 2001 |  | 9.66 | 9.27 |  | 306.14 | 306.94 |  | 49.65 | 48.56 |  | 13.18 | 13.54 |  | 27.98 | 41.76 |  | / | / |  | 5.57 | 12.15 |  | 0.71 | 3.63 |
| Bosnia & Herzegovina ^e^ | / |  | 2.93 | 3.28 |  | 35.52 | 15.03 |  | 37.00 | 39.11 |  | 2.49 | 5.22 |  | / | / |  | / | / |  | 0.05 | 0.44 |  | / | 1.10 |
| Hungary ^e^ | / |  | 7.58 | 6.52 |  | 153.42 | 149.22 |  | 38.49 | 41.84 |  | 15.00 | 12.60 |  | 15.42 | 10.51 |  | 0.60 | / |  | 2.43 | 2.28 |  | 6.36 | 7.40 |
| Kazakhstan ^e^ | / |  | 21.05 | 13.84 |  | 46.48 | 45.69 |  | 85.13 | 86.16 |  | 18.52 | 17.73 |  | / | 16.1 |  | 1.01 | / |  | 12.51 | 16.71 |  | / | 26.10 |
| Russia ^e^ | / |  | 29.62 | 21.23 |  | 67.70 | 64.06 |  | 52.87 | 48.00 |  | 24.60 | 18.11 |  | 29.8 | 11.1 |  | 13.00 | 10.40 |  | 57.20 | 58.43 |  | 28.80 | 19.50 |

*Notes*. As we only have sales data from 2014Q2 until 2020Q1, we report the value three quarters before and four quarters after the switch of Germany, Poland and Italy for all available countries. For other outcomes we report the value two years prior and two years post intervention. Abbreviations: EC, emergency contraception; RC, regular contraception

^a^ Sold units per 1000 women aged 15-49

^b^ Months of achieved birth control by regular contraceptives per 1000 women aged 15-49

^c^ Rates per 1000 women aged 15-49, yearly data.

^d^ Incidence rates per 100.000 citizens; yearly data.

^e^ As Bosnia & Herzegovina, Hungary, Kazakhstan and Russia were never treated we refer to 2015 as base year for t-2 and t+2.

^f^ t-1 instead of t-2 (1998).

| **10. Results including country specific linear time trends**  **Table A13** Regression results OTC switch with country-specific linear time trends | | | | | |
| --- | --- | --- | --- | --- | --- |
|  | TWFE Modell III (Covariates, Wboot) + CSLTT | | | | |
|  | OTC switch | |  | Rx switch | |
| Use |  |  |  |  |  |
| Emergency contraception | 2.548 | * |  | -4.107 |  |
|  | [0.500; 4.445] |  |  | [-12.495; 2.147] |  |
| Regular contraception | 0.054 |  |  | 11.703 |  |
|  | [-3.830; 5.194] |  |  | [-46.126; 56.842] |  |
| Demographic outcomes |  |  |  |  |  |
| Birth rate | 0.387 |  |  | -0.038 |  |
|  | [-1.053; 1.904] |  |  | [-0.611; 0.601] |  |
| Abortion rate | -0.259 |  |  | -0.312 |  |
|  | [-0.741; 0.235] |  |  | [-6.001; 4.958] |  |
| Health outcomes |  |  |  |  |  |
| Gonorrhoea | -0.694 |  |  | -6.521 |  |
|  | [-2.993; 1.561] |  |  | [-74.715; 48.783] |  |
| Hepatitis B | -1.814 |  |  | 2.316 |  |
|  | [-3.709; 0.127] |  |  | [-12.749; 17.011] |  |
| HIV | 0.282 |  |  | 0.127 |  |
|  | [-0.471; 1.025] |  |  | [-13.710; 11.606] |  |
| Syphilis | 0.055 |  |  | 1.483 |  |
|  | [-2.240; 2.612] |  |  | [-11.738; 14.767] |  |

*Notes*: p<0.1 (*). 90% confidence intervals are shown in brackets. TWFE model III (see main paper) including country specific linear time trends. Emergency contraception sales in IQVIA MIDAS standard units / 1000 women aged 15-49. Regular contraception sales in months of achieved birth control by regular contraceptives per 1000 women aged 15-49. Births are measured in annually live births per 1000 fertile women. Abortions are measured in yearly abortions per 1000 fertile women. STIs are measured in incidence rate (per 100,000 population). Abbreviations: OTC, over-the-counter; CSLTT, country-specific linear time trends; TWFE, Two-way fixed effects.

**11. Event study plots (Rx switch)**

**Figure A3** Event study plots (Rx switch).

Notes**.** 95% confidence intervals. TWFE model including covariates and wild cluster bootstrapping (10,000 replications). EC use in IQVIA MIDAS standard units / 1000 fertile women. RC use in QSU / 1000 fertile women (see methods section for QSU definition). Births/abortion rates per 1,000 fertile women.

**11. SDID results before/after the Rx switch of Poland**

**Figure A4** Outcome trends of the SDID estimator by Arkhangelsky et al (2021) before/after the Rx switch of Poland*.*

*Notes*. The vertical line represents the date of Poland’s switch back to prescription bound status, i.e. the first treated point in time. The *Treated* group refers to Poland. *Control* refers to the weighted synthetic control group created by the SDID estimator. Emergency contraception use in IQVIA MIDAS standard units / 1000 fertile women. Regular contraception use in QSU / 1000 fertile women (see methods section for QSU definition). Births are measured in monthly live births per 1000 fertile women. Abortions are measured in annual abortions per1000 fertile women. STIs are measured in annual incidence rate (per 100,000 population). The Synthetic DiD model includes the same covariates as TWFE model II and III (GDP per capita, female rate in total population, abortion laws).

**Figure A5** Synthetic control groups of the SDID estimator by Arkhangelsky et al (2021)*.*

*Notes*. The y-axis reports the individual country-by-country adjusted difference in outcomes. The horizontal line represents the estimated effect (weighted average of the adjusted differences). The relative weights are indicated by the size of the markers. Weights of zero are indicated with a *x*. Emergency contraception use in IQVIA MIDAS standard units / 1000 fertile women. Regular contraception use in QSU / 1000 fertile women (see methods section for QSU definition). Births are measured in monthly live births per 1000 fertile women. Abortions are measured in annual abortions per 1000 fertile women. STIs are measured in annual incidence rate (per 100,000 population). The Synthetic DiD model includes the same covariates as TWFE model II and III (GDP per capita, female rate in total population, abortion laws).

**12. Additional analysis of fertility**

In order to increase comparability, we also carried out the analyses that we performed for the birth rate for fertility.

| **Table A14** Regression results OTC switch: Fertility | | | | | | | | |
| --- | --- | --- | --- | --- | --- | --- | --- | --- |
|  | TWFE Results | | | | | | Robustness Check | |
|  | (I):  Basic Model | | (II):  + Covariates | | (III):  + Covariates  + Wboot | | dC & D'H (2020b) ^a^ | |
| Fertility | 0.007 |  | 0.030 |  | 0.030 |  | -0.030 |  |
|  | [-0.085; 0.100] |  | [-0.054; 0.113] |  | [-0.054; 0.112] |  | [-0.107; 0.046] |  |

*Notes*: p<0.1 (*), p<0.05 (**), p<0.01 (***). TWFE models I and II with cluster robust standard errors. 90% confidence intervals are shown in brackets. Modell II includes the GDP per capita, the share of females in the total population and abortion laws as covariates. Model III includes more conservative wild t-bootstrap standard errors (1000000 reps). Abbreviations: OTC, over-the-counter; TWFE, Two-way fixed effects; Wboot, Wild cluster bootstrapping; dC & D'H, de Chaisemartin & D’Haultfoeuille

^a^ 'DID_M' - estimator by de Chaisemartin & D'Haultfoeuille (2020a); Block bootstrapped standard errors at country level (reps: 1000). Without controls as the number of control variables exceeds the sample size in some bootstrap replications, which would return noisy estimates (especially for sales/use regressions). The table reports the difference between the average number of treatment units’ first-time switchers received after their first switch and the number of treatment units they would have received if they had never switched is used to scale the average of the periodical effects computed by the estimator by de Chaisemartin & D'Haultfoeuille (2020b). Please see their paper for more details.

| **Table A15** Regression results Rx switch: Fertility | | | | | | | | |
| --- | --- | --- | --- | --- | --- | --- | --- | --- |
|  | TWFE Results | | | | | | Robustness Checks | |
|  | (I):  Basic Model | | (II):  + Covariates | | (III):  + Covariates + Wboot | | Synth. DiD ^a^ | |
| Fertility | 0.060 |  | 0.034 | * | 0.034 |  | -0.039 |  |
|  | [-0.001; 0.122] |  | [0.002; 0.065] |  | [-0.323; 0.444] |  | [-0.129; 0.052] |  |

*Notes*: p<0.1 (*), p<0.05 (**), p<0.01 (***). 90% confidence intervals are shown in brackets. TWFE models I and II with cluster robust standard errors. Modell II includes the GDP per capita, the share of females in the total population and abortion laws as covariates. Model III includes more conservative wild t-bootstrap standard errors (1000000 reps). Abbreviations: OTC, over-the-counter; TWFE, Two-way fixed effects; Wboot, Wild cluster bootstrapping; SDID, Synthetic difference-in-differences.

^a^ Synthetic DiD by Arkhangelsky et al (2021). We only include time periods after Poland's initial OTC switch in order to compare only OTC periods before the Rx switch ( 2015 - 2021). The Synthetic DiD model includes the same covariates as TWFE model II and III (GDP per capita, female rate in total population, abortion laws).

**Figure A6** Event study plots (OTC switch): Fertility

*Notes.* Plots were created with event_plot Stata module by Borusyak et al. [1]. 90% confidence intervals. De Chaisemartin & D'Haultfoeuille (2020b) estimator without control variables as otherwise the number of control variables in the event study exceeded the sample size in some bootstrap replications, which would return too noisy estimates (especially for sales/use regressions).

**Figure A7** Outcome trend of the SDID estimator by Arkhangelsky et al (2021) before/after the Rx switch of Poland*.* : Fertility

*Notes*. The vertical line represents the date of Poland’s switch back to prescription bound status, i.e. the first treated point in time. The *Treated* group refers to Poland. *Control* refers to the weighted synthetic control group created by the SDID estimator.

| **Table A16** Data sources for fertility | | | |
| --- | --- | --- | --- |
| Country | Fertility ^a^ | Country | Fertility |
| Austria | 1998-2021: United Nations | Kazakhstan | 1998-2021: World Bank ^b^ |
| Belgium | 1998-2021: United Nations | Latvia | 1998-2021: United Nations |
| Bosnia and Herzegovina | 1998-2021: World Bank ^b^ | Lithuania | 1998-2021: United Nations |
| Bulgaria | 1998-2021: United Nations | Luxembourg | 1998-2021: United Nations |
| Croatia | 1998-2021: United Nations | Malta | 1998-2021: United Nations |
| Czech Republic | 1998-2021: United Nations | Netherlands | 1998-2021: United Nations |
| Denmark | 1998-2021: United Nations | Norway | 1998-2021: United Nations |
| Estonia | 1998-2021: United Nations | Poland | 1998-2021: United Nations |
| Finland | 1998-2021: United Nations | Portugal | 1998-2021: United Nations |
| France | 1998-2021: United Nations | Russia | 1998-2021: World Bank ^b^ |
| Germany | 1998-2021: United Nations | Slovakia | 1998-2021: United Nations |
| Greece | 1998-2021: United Nations | Slovenia | 1998-2021: United Nations |
| Hungary | 1998-2021: United Nations | Spain | 1998-2021: United Nations |
| Iceland | 1998-2021: United Nations | Sweden | 1998-2021: United Nations |
| Ireland | 1998-2021: United Nations | Switzerland | 1998-2021: United Nations |
| Italy | 1998-2021: United Nations | United Kingdom | 1998-2021: United Nations |

*Notes:*

^a^ United Nations, Department of Economic and Social Affairs, Population Division (2022). World Population Prospects: The 2022 Revision

^b^ World Bank: World Development Indicators. <https://databank.worldbank.org/source/world-development-indicators/preview/on> (Indicator codes: SP.POP.TOTL, SP.POP.1014.FE.5Y, SP.POP.1519.FE.5Y, SP.POP.2024.FE.5Y, SP.POP.2529.FE.5Y, SP.POP.3034.FE.5Y, SP.POP.3539.FE.5Y, SP.POP.4044.FE.5Y, SP.POP.4549.FE.5Y, SP.POP.TOTL.FE.IN, SP.DYN.TFRT.IN, SP.DYN.CBRT.IN )

| **TableA17** Descriptive statistics: Fertility | | | | | |
| --- | --- | --- | --- | --- | --- |
|  | *N* | *Mean* | *Mean (pre)* | *Min* | *Max* |
| *Fertility ^a^* | 768 | 1.57 | 1.45 | 1.12 | 3.32 |

*Notes*. The theoretically possible maximum number of observations between 1998 and 2021 is 768 (32x 24 years). Mean, minimum and maximum values are measured across all countries and observed years/quarters. Mean (pre) only includes pre-intervention periods of treated countries.

^a^ Mean total fertility per year between 1998 and 2021. The total fertility rate in a specific year is defined as the total number of children that would be born to each woman if she were to live to the end of her child-bearing years and give birth to children in alignment with the prevailing age-specific fertility rates (OECD); yearly data.

| **Table A18** Included (pre-intervention) periods: Fertility | | | | | | | | | |
| --- | --- | --- | --- | --- | --- | --- | --- | --- | --- |
|  | OTC-switch | | | |  | Rx-switch | | | |
| Outcome | Total observed time span | Pre-intervention time span | Measure | Pre-periods |  | Total observed time span | Pre-intervention time span | Measure | Pre-periods |
| Fertility | 1998 – 2017 | 1998 – 2015  (lagged) | Years | 18 |  | 2016 – 2021 | 2016 – 2017  (lagged) | Years | 2 |

*Notes*. Observed periods that are included in each analysis.

| **Table A19** Country selection depending on intervention and outcome: Fertility | |
| --- | --- |
| Outcome | Countries |
| OTC |  |
| Fertility | Full sample (all 32 countries)  Intervention countries: Germany, Italy, Poland, Croatia |
| Rx (TWFE DID) |  |
| Fertility | Intervention countries: Poland  Control countries: Germany, Italy, Croatia, Hungary, Bosnia and Herzegovina, Russia, Kazakhstan, France, Norway, Portugal, United Kingdom, Belgium, Sweden ^a^ |
| Rx (SDID) |  |
| Fertility | Full sample except always-treated countries (Hungary, Russia, Kazakhstan, Bosnia and Herzegovina)  Intervention countries: Poland |

*Notes*: Synthetic DID does not support unbalanced data (i.e. missing values) or always treated countries. In case of Poland’s Rx switch, countries that have never facilitated OTC access to emergency contraceptives before are considered as ‘always-treated countries’. Abbreviations: OTC, over-the-counter; TWFE, Two-way fixed effects; SDID, Synthetic difference-in-differences.

^a^ All treated countries within the observed time span, all never-treated countries, oldest always-treated countries. Birth rate without Bosnia and Herzegovina, Kazakhstan and Russia due to non-availability of monthly data.

| **Table A20** Potential confounders' attributes: Fertility | | | | | | | |
| --- | --- | --- | --- | --- | --- | --- | --- |
| Outcome | Variation in time |  | Relation of variation in time to intervention |  | Marginal effect on the outcome |  | Adjustment |
| Outcome: Fertility |  |  |  |  |  |  |  |
| GDP per Capita | Time-varying |  | Independent of intervention |  | Time-varying |  | X*time |
| Life expectancy | Time-varying |  | Independent of intervention |  | Time-invariant |  | None |
| Population density | Time-varying |  | Independent of intervention |  | Time-invariant |  | None |
| Unemployment rate | Time-varying |  | Independent of intervention |  | Time-invariant |  | None |
| Women share (population) | Time-varying |  | Independent of intervention |  | Time-varying |  | X*time |
| Abortion law | Time-varying |  | Independent of intervention |  | Time-varying |  | X*time |

*Notes*: Based on the framework by Zeldow and Hatfield (2021).
